# How Government Policies and Organisational and Sectoral Circumstances Influence Nurse Practitioner and Physician Assistant Employment and Training: A Realist Analysis Using Surveys

**DOI:** 10.1111/jan.70433

**Published:** 2025-12-15

**Authors:** Ellen J. C. M. Dankers‐de Mari, Anneke J. A. H. van Vught, Patrick P. T. Jeurissen, Ronald Batenburg

**Affiliations:** ^1^ Radboud Institute for Health Sciences, Scientific Department (IQ Health) Radboud University Medical Center Nijmegen the Netherlands; ^2^ Advisory Committee on Health Workforce Planning Capaciteitsorgaan Utrecht the Netherlands; ^3^ HAN University of Applied Sciences Nijmegen the Netherlands; ^4^ Dutch Healthcare Authority Utrecht the Netherlands; ^5^ Netherlands Institute for Health Services Research NIVEL Utrecht the Netherlands; ^6^ Radboud University Nijmegen Nijmegen the Netherlands

**Keywords:** evaluation study, government, health workforce, healthcare policy, nurse practitioners, physician assistants, physician associates

## Abstract

**Aims:**

To explain how government policies affected decision‐making on Nurse Practitioner and Physician Assistant employment and training within Dutch healthcare organisations, and how organisational and sectoral circumstances were influential.

**Design:**

An online, cross‐sectional survey study.

**Methods:**

A literature‐ and interview‐based program theory was tested using surveys. Respondents from hospital care, (nursing) home care, primary care, and intellectual disability services were recruited using convenience sampling. Data analysis used descriptive statistics and inferential tests. Open‐ended responses were analysed using thematic synthesis techniques. Survey results were clustered to assess verification, falsification, or refinement of program theory elements.

**Results:**

A total of 568 experts in hiring and training healthcare professionals participated. Respondents indicated that most government policies promoted employment and training. Organisational and sectoral circumstances caused significant variations in Nurse Practitioner and Physician Assistant deployment across healthcare sectors, shaping how decision‐makers interpreted and acted on government policies. Specific circumstances within primary care hampered deployment.

**Conclusion:**

Government policies stimulated training and employment by: (1) removing practice restrictions (scope of practice expansion, legal acknowledgment), (2) facilitating cost‐effective training and deployment (training grants, billing options), (3) providing sectoral knowledge on deployment, training, and healthcare outcomes (funding research and a sectoral knowledge center), and (4) establishing sectoral agreements (on apprenticeships). Organisational and sectoral circumstances significantly influenced outcomes. Key circumstances included flanking policies, stakeholder support, labor market capacity, healthcare demand, organisational resources and aims, and type of decision‐makers (medical doctor or manager/director). Familiarity with the professions stimulated deployment.

**Impact and Implications:**

The refined and verified program theory supports designing effective skill‐mix policies and facilitating Nurse Practitioner and Physician Assistant employment and training. Tailoring skill‐mix policies can optimise outcomes. This offers opportunities for governments, healthcare funders, organisations, and professionals to contribute to healthcare quality, cost efficiency, and patient satisfaction.

**Patient or Public Contribution:**

Healthcare professionals were part of the study population.

## 1 Introduction

Governments worldwide seek solutions for rising healthcare demand amid financial and personnel shortages (Azzopardi‐Muscat et al. 2023; Valentin et al. 2021). Task shifting to Nurse Practitioners (NPs) and Physician Assistants (PAs) is considered one potential solution. NPs and PAs can take over tasks from medical doctors, providing the same quality of care and higher patient satisfaction at lower or comparable costs (Halter et al. 2018; Laurant et al. 2018; Liu et al. 2020; van den Brink et al. 2021). Approximately 40 countries have well‐established advanced practice nursing roles, including NPs and clinical nurse specialists, while PAs or PA‐comparable professions exist in over 50 countries (Showstark et al. 2021; Wheeler et al. 2022).

Despite the interest of governments in task shifting to NPs and PAs, limited research has examined how government policies concerning task shifting impact the actual deployment of NPs and PAs within healthcare organisations. Studies in the USA indicated that: (1) expanding the scope of practice increased NP and PA deployment, while reimbursement policies and financial incentives posed barriers (Ainslie et al. 2024; Kuo et al. 2013; O'Reilly‐Jacob et al. 2024; Patel et al. 2019; Poghosyan, Nannini, Smaldone, et al. 2013; Spetz et al. 2017; Valentin et al. 2021; Xue et al. 2018). UK studies indicated that a lack of practice regulation, oversight, prescribing authority, and evidence on NP/PA inclusion contributed to uncertain employment prospects (Drennan et al. 2023; Halter and Wheeler 2017; McKee et al. 2025). This was supported by a European study showing greater task shifting in countries with legislative and regulatory change (Maier et al. 2018).

## 2 Background

In the Netherlands, a comprehensive program of NP/PA policies has been implemented (Table 1). The country has one of the highest numbers of NPs in Europe, alongside a higher‐than‐average number of nurses and a lower‐than‐average number of physicians compared with other European Union countries. The Netherlands has also increasingly deployed PAs to support doctors (OECD/European Commission 2024).

**TABLE 1:** Resume of NP/PA government policies in the Netherlands 2000–2019.

Training grants^a^	The master's‐level training mainly occurs within the healthcare organisation employing the trainee, with one day per week of in‐school training. Grants cover salary costs for trainee replacement during the course and the in‐school program. Primary care providers can apply for additional funding. Policy aims include improving care quality, enhancing career prospects, and preventing staff shortages
Reimbursement regulations^a^	NPs and PAs have expanded options to register and bill for healthcare services. Since 2015, they can independently open and close tasks in the hospital and rehabilitation care reimbursement systems without mandatory face‐to‐face doctor‐patient contact. In 2019, they were allowed to bill for peer consultations and co‐treatment in hospital care. Additionally, from 2020, NPs and PAs in (nursing) home care and intellectual disability services can provide and bill for extramural care for vulnerable groups in primary care
Knowledge and consultation platforms and research^a^	The Dutch government funded knowledge and consultation platforms and (evaluation) research on task shifting. In 2004, it funded the Consultation Platform NP and PA, the Steering Committee Task shifting in Primary Care, and the National Center of Knowledge for Task shifting in Primary Care
Scope of practice^a^	Since 2012, NPs and PAs have been authorised to perform certain reserved medical procedures. As of September 1, 2018, their autonomous scope of practice became definitive, allowing them to independently indicate, execute, and delegate various reserved medical procedures depending on experience and specialty. These include surgical procedures, endoscopies, catheterizations, injections, punctures, elective cardioversion, defibrillation, and prescribing prescription‐only drugs
Legal acknowledgment professional and educational level^a^	Since 2009 and 2018, the NP and PA professions are included in the Individual Health Care Professions Register and Act, granting them a protected title and disciplinary governance. From January 1, 2014, and September 1, 2016, NP and PA graduates receive an MSc degree

Abbreviations: NP, nurse practitioner; PA, physician assistant.

^a^ A more detailed description of the policy measures can be found in Dankers‐de Mari, van Vught, et al. (2023).

Internationally, NPs are defined as generalist nurses with at least a master's‐level education who provide autonomous care, following evidence‐informed guidelines and focusing on the whole person rather than only the condition or disease (International Council of Nurses 2020). PAs are classified as paramedical practitioners who provide medical services more limited in scope and complexity than those carried out by medical doctors, working either autonomously or under limited supervision (International Labour Office 2012). Across countries, the level of practice autonomy and activities of NPs and PAs vary, reflecting contextual and regulatory differences (International Council of Nurses 2020; Showstark et al. 2023). In the Netherlands, PAs and NPs are autonomously authorized to perform specific reserved medical procedures. Dutch PAs work within a specific medical domain, with an emphasis on the medical model, while NPs integrate nursing and medical care, focusing on the biopsychosocial model and care coordination (Laurant and van Vught 2018).

The NP and PA professions entered the Dutch labor market in 2000 and 2004, respectively. In January 2025, 5932 NPs and 2354 PAs were registered (CIBG 2024). From 2013 to 2022, NP and PA full‐time equivalents (FTEs) grew annually by 14% and 16%, outpacing specialised medical doctors (2%). Relatively many NPs worked in mental healthcare and (nursing) home care (residential/nursing home and home‐based nursing care), with 42 and 41 FTEs per 100 FTEs of specialised medical doctors. The proportion of NPs working in hospital care (including rehabilitation centers and ambulance care) and primary care settings (general practices and out‐of‐hours services) was lower, at 9 and 3 FTEs for NPs and 6 and 2 FTEs for PAs, respectively. The largest proportions of NPs and PAs were employed in hospital care, accounting for 42% and 77%, respectively. Since 2004, intake into NP and PA training programs has greatly increased, with significant variation across sectors (Dankers‐de Mari, van Vught, et al. 2023).

**FIGURE 1:**
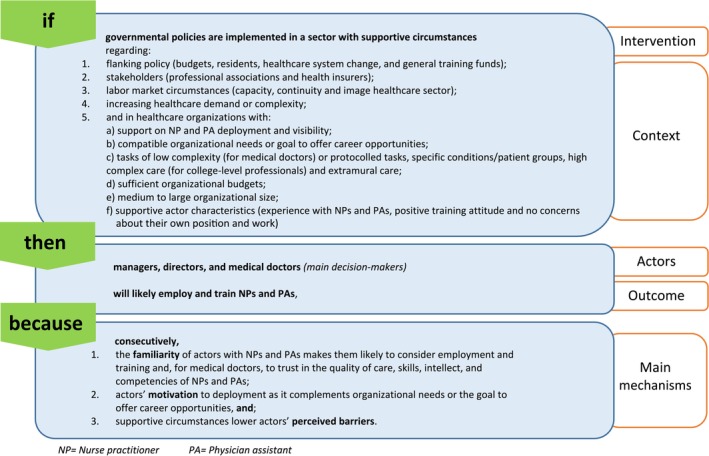
Refined program theory.

Dankers‐de Mari, Thijssen, et al. (2023) presented a refined program theory based on literature and 50 interviews, showing how Dutch NP/PA government policies and circumstances influenced deployment within healthcare organisations in non‐mental healthcare sectors (Figure 1). Policies enhanced employment and training through three mechanisms: (1) contributing to familiarity and trust (sectoral task shifting knowledge center), (2) activating motivation (scope of practice expansion and reimbursement regulations), and (3) clearing perceived barriers (training grants, scope of practice expansion, legal acknowledgment, and reimbursement regulations).

However, these results also showed significant differences in NP and PA deployment and training across sectors. This indicates that the impact of NP/PA policies was strongly determined by sectoral and organisational circumstances, such as flanking policies, stakeholder support, labor market conditions, healthcare demand and healthcare setting characteristics. This aligns with international findings that organisational circumstances are at least as important as legal scope of practice barriers in determining how NPs and PAs are utilised (Pittman et al. 2020; Pittman et al. 2021; Poghosyan, Nannini, Stone, and Smaldone 2013; Yee et al. 2013). To clarify why government policies have varying effects across sectors, further refinement and testing of the refined program theory at the sectoral level—particularly regarding decision‐makers and circumstances—is needed.

## 3 The Study

This study aimed to further refine and test a refined program theory on how government policies influence the healthcare organisations' decision‐making processes about NP and PA employment and training, and how organisational and sectoral circumstances exert influence. The refined program theory is based on the realist evaluation approach, where the premise is that whether interventions work depends on how actors respond to what is offered in the intervention. This interaction between the intervention and actors in specific contexts triggers mechanisms that lead to certain outcomes (Marchal et al. 2019). We tested the refined program theory across a broad, diverse group of decision‐making experts to explain variation in NP and PA deployment and training across healthcare sectors. We addressed the following overarching research question: ‘How do the survey results align with the refined program theory?’ To answer this, we examined the following sub‐questions, based on Dankers‐de Mari et al. (2021):

1. How was the decision‐making process regarding the employment and training of NPs and PAs structured within healthcare organisations, and who were the participants involved? (actors)
2. How did participants in the decision‐making process within healthcare organisations interpret and act upon governmental policies? (mechanisms)
3. Which circumstances affected participants' decision‐making regarding NPs' and PA's employment and training? (context)
4. How and why did governmental policies contribute to NP and PA employment and training, for whom, under what circumstances, and in what respects?

## 4 Methods

### 4.1 Design

We conducted a cross‐sectional survey study among Dutch healthcare organisations to examine refined program theory elements, focusing on: (1) completeness, (2) frequency of occurrence, and (3) sectoral differences.

### 4.2 Data Collection

Data were collected in July and August 2019 through a research project initiated by the Dutch Advisory Committee on Health Workforce Planning.

### 4.3 Study Setting and Sampling

The survey was distributed among four sectors, namely (1) hospital care (including rehabilitation and ambulance services), (2) primary care, (3) (nursing) home care, and (4) intellectual disability services. Respondents were self‐identified experts from healthcare organisations with knowledge of the decision‐making processes regarding hiring and training healthcare professionals. These included directors, CEOs, managers, specialised medical doctors, practical trainers, Human Resources professionals, and educational advisors.

The method of engagement involved email invitations distributed through sectoral and professional associations, colleges, and the network of the research firm that launched the survey. Members and contacts were invited to complete the questionnaire, followed by a reminder. To enhance the response rate, the invitation emphasised that the study results would contribute to advice to the Ministry of Health regarding the required training capacity for healthcare professionals. Additionally, respondents were informed that participation was anonymous and that they could win a dinner voucher. The distribution lists included healthcare organisations and professionals. Furthermore, news reports and social media posts were published to raise awareness of the study.

### 4.4 Instruments

Seven subsector‐specific surveys were developed to assess factors influencing NP and PA employment and training, focusing on national policies, funding, and labor market conditions. The questionnaires were based on the initial program theory (Dankers‐de Mari et al. 2021) and interviews with professionals working in these healthcare sectors (Dankers‐de Mari, Thijssen, et al. 2023). Surveys were adjusted and validated by feedback from the Advisory Committee on Health Workforce Planning PA/NP chamber, consisting of training program coordinators, NPs, PAs, and health insurer representatives. Each survey consisted of the same sections (Box 1).

### 4.5 Exclusion Criteria

Respondents were excluded if they had no insight into the decision‐making regarding NPs and PAs.

### 4.6 Data Analysis

Data were analyzed using SPSS Statistics (version 29). Descriptive statistics were calculated for all variables, and chi‐square and Fisher–Freeman–Halton Exact tests were conducted to explore significant differences between sectors and respondent types. A *p*‐value of < 0.05 was considered statistically significant.

For the calculation of descriptive statistics, all cases for each variable were used. Survey results were clustered in line with refined program theory elements to assess whether they correspond (verification), deviate (falsification), or supplement these elements (refinement). Open‐ended responses were labelled according to refined program theory elements (deduction). Where no refined program theory element was applicable, new themes were created (induction). During the analysis, it was determined that falsification of refined program theory elements was not possible based on the available data, as (1) respondents were asked to select the reasons and barriers they considered most important, rather than those they deemed inapplicable, and (2) none of the refined program theory elements were entirely unselected or unmentioned. Therefore, the focus of the analysis shifted toward refinement and verification.

Data synthesis focused on unravelling how similar policies led to different deployment and training outcomes across healthcare sectors. For this, we used retroduction—a central inference‐making method in realist research (RAMESES 2017). Retroductive theorising involves starting with a program's effects and working backward to consider the conditions necessary for such effects to manifest (Jagosh 2020; Mukumbang et al. 2021). It is a conceptualization method to identify the circumstances without which a phenomenon cannot occur (Meyer and Lunnay 2013; Mukumbang et al. 2021). The findings were further examined through subgroup comparison (Pawson and Tilley 2004) at the sectoral level. A configuration matrix served as a heuristic tool, incorporating elements of the refined program theory (Intervention, Context, Actor, Mechanisms) and using sectoral Outcomes as the analytical starting point (Mukumbang et al. 2019; Pawson and Tilley 1997). For interpretation of the survey results, we built on the causal relationships and mechanisms previously identified by Dankers‐de Mari, Thijssen, et al. (2023). Distinct sectoral circumstances and decision‐making process participants were identified to explain variation in NP and PA employment and training across sectors.

BOX 1 **Sections Questionnaires.**

(A) work setting (e.g. organisation type, department, location), number of NP/PAs, insight into the decision‐making of employment/training; (B) initiators in the decision‐making process; (C) decision‐makers; (D) influencers; (E) supporters; (F) type of employer (hospital and/or physician‐led company); (Q) main reasons and incentives for employment and training; (R) main barriers to employment and training; (S) expectations about future deployment and influencing factors; (T) respondent characteristics (position, medical specialism, municipality, number of locations, and number of employees of the healthcare organisation).

### 4.7 Ethical Considerations

The Ethics Committee on Human Subjects Research Radboudumc waived the need for ethics approval. The study was not eligible for assessment because there were no indications that: (1) participants would experience participation in the research as overly burdensome given their condition or the nature of the research; and (2) the research would generate previously unknown data about the (future) health status of a participant or blood relatives (Radboudumc 2022). Informed consent was obtained. No personal data were collected. Data were stored securely in a data repository (Dankers‐de Mari et al. 2025). The survey was anonymous and no identifiable information was collected.

This study is reported in accordance with the CROSS (Sharma et al. 2021) and RAMESES (RAMESES 2017) guidelines.

## 5 Results

### 5.1 Characteristics of the Sample

**FIGURE 2:**
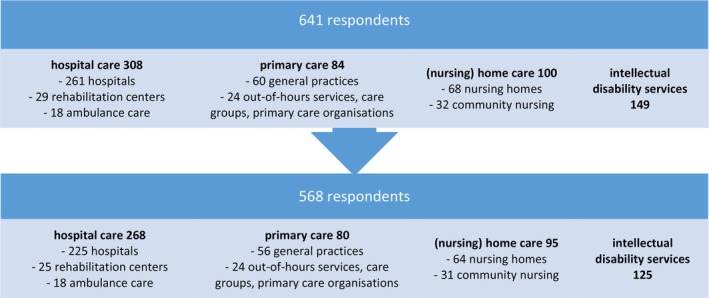
Respondents per sector.

Of the 6469 people approached, 2509 opened the introduction and sector selection page, 1041 selected a sector, and 641 fully completed a subsector questionnaire, which resulted in a response rate of 10%. 568 respondents were included in the analysis after applying exclusion criteria. Figure 2 shows the number of respondents in total and per sector. Table 2 presents respondents' roles within each sector. Managers and directors represented the largest group of respondents in all sectors, except for primary care, where medical doctors constituted the majority.

**TABLE 2:** Respondents per role and sector (*N* = 568).

	Hospital care^a^	Primary care^a^	(Nursing) home care^a^	Intellectual disability services^a^	Total^a^
Management/(board of) directors	52%	48%	64%	54%	54%
Specialised medical doctors	31%	51%	17%	14%	28%
Human Resources professionals	9%	5%	5%	22%	11%
Educational advisors/managers	18%	6%	2%	6%	11%
NP/PA professional or working group	6%	0%	7%	6%	5%
Nursing staff/nursing advisory council	1%	0%	4%	5%	2%
Other	3%	1%	13%	8%	6%

Abbreviations: NP, nurse practitioner; PA, physician assistant.

^a^ Percentages do not add up to 100% because respondents could select multiple roles.

**FIGURE 3:**
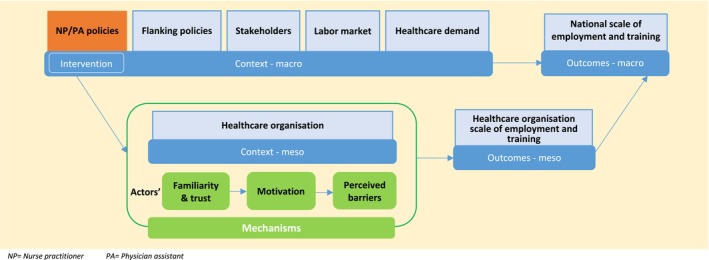
Refined and verified Intervention‐Context‐Actor‐Mechanism‐Outcome configurations.

**FIGURE 4:**
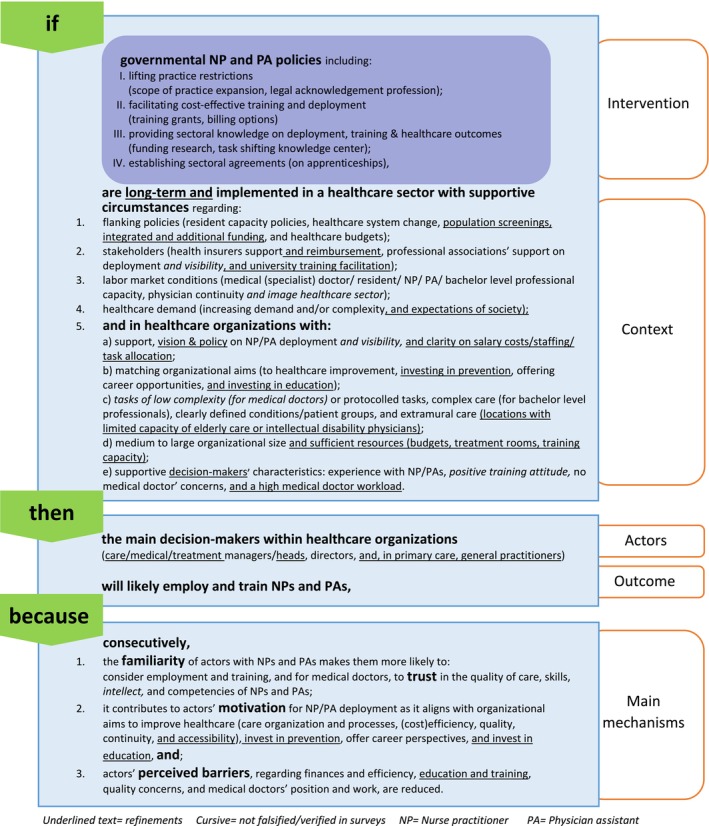
Detailed refined and verified program theory.

Results are presented in relation to the refined program theory on how government NP/PA policies (i.e., *the intervention*) affected the number of NPs and PAs working and training in Dutch healthcare (*outcome*) as a result of how organisational and sectoral circumstances (*context*) influenced decision‐making participants within healthcare organisations (*actors*) and how policies and circumstances were interpreted and acted upon (*mechanisms*). The Intervention‐Context‐Actor‐Mechanism‐Outcome configurations in Figure 3 show how these components were related, illustrating how, why, and under what circumstances policies affected participants in the decision‐making process. We assessed which components of the refined program theory were verified or supplemented, and we describe patterns that explain sectoral differences in deployment and training outcomes between sectors. We present the overall insights in a refined and verified program theory (Figure 4). A more detailed version of Figure 3 including verifications and refinements can be found in Appendix S1.

In our survey, respondents mentioned various stimuli and barriers to NP and PA employment and training (Tables 3 and 4). The primary stimuli were: (1) healthcare improvement, (2) the labor market, and (3) changing healthcare demands. There were significant differences between sectors. Respondents in primary care less frequently indicated ‘healthcare improvement’ as a stimulus, hospital care respondents less frequently mentioned ‘labor market’, and (nursing) home care and intellectual disability services respondents less frequently cited ‘funding’.

**TABLE 3:** Main reasons/stimulating factors for hiring and/or training PAs and/or NPs within the own work setting (up to 3 answers).

	Hospital care		Primary care		(Nursing) home care		Intellectual disability services		Total				
	*n*	%	*n*	%	*n*	%	*n*	%	*n*	%	*χ* ^2^	df	*p*
Healthcare improvement	168	83	29	62	54	75	45	79	296	78	10.393	3	0.016*
Healthcare professionals' labor market	106	52	38	81	51	71	37	65	232	61	17.689	3	0.001**
Changing healthcare demand	75	37	26	55	30	42	27	47	158	42	6.227	3	0.101
Funding	67	33	15	32	11	15	9	16	102	27	12.969	3	0.005**
Familiarity/positive experiences	48	24	7	15	17	24	10	18	82	22	2.472	3	4.80
Policy of governments, umbrella organisations or healthcare organisations	27	13	4	9	12	17	6	11	49	13	2.026	3	0.567
Education and training	15	7	4	9	5	7	4	7	28	7	0.119	3	0.990
Total number of respondents	203		47		72		57		379				

Abbreviations: NP, nurse practitioner; PA, physician assistant.

* < 0.05; ** < 0.01.

**TABLE 4:** Main barriers to hiring and/or training PAs and/or NPs within the own work setting (up to 3 answers).

	Hospital care		Primary care		(Nursing) home care		Intellectual disability services		Total				
	*n*	%	*n*	%	*n*	%	*n*	%	*n*	%	*χ* ^2^	df	*p*
Funding	90	41	29	48	13	16	19	17	151	32	36.781	3	0.000**
Lack of familiarity with the professions	38	18	13	21	16	20	36	32	103	22	9.186	3	0.027*
No need for change	37	17	9	15	13	16	28	25	87	18	4.098	3	0.251
Healthcare professionals' labor market	20	9	9	15	10	13	28	25	67	14	14.985	3	0.002**
Practical	27	12	19	31	9	11	12	11	67	14	16.666	3	0.001**
Quality of care	30	14	7	11	8	10	6	5	51	11	5.665	3	0.129
Education and training	32	15	14	23	14	18	13	12	73	15	4.302	3	0.231
Policies of governments, umbrella organisations, and healthcare organisations	10	5	6	10	5	6	8	7	29	6	2.497	3	0.476
Changing healthcare demand	10	5	3	5	3	4	5	4	21	4	0.136	3	0.987
Not applicable/don't know	44	20	12	20	26	33	19	17	101	21	7.532	3	0.057
Total number of respondents	217		61		80		113		471				

Abbreviations: NP, nurse practitioner; PA, physician assistant.

* < 0.05; ** < 0.01.

Primary barriers included: (1) funding, (2) unfamiliarity with NPs and PAs, and (3) perceived lack of need for change. In intellectual disability services, ‘unfamiliarity with the professions’ and ‘labor market’ were significantly more mentioned. Funding issues were cited more commonly in hospital and primary care, as compared to (nursing) home care and intellectual disability services. Further, practical barriers were more frequently mentioned in primary care.

Subsequently, we discuss the survey results in relation to the refined program theory. The numbers and percentages of follow‐up and additional questions are provided in Appendix S2. Percentages are based on the number of respondents that answered questions. For questions with 10 or fewer respondents, absolute numbers are displayed.

### 5.2 How Policies and Circumstances Were Interpreted and Acted Upon (Mechanisms)

Mechanisms describe how decision‐making participants interpreted and acted upon policies and circumstances, and how this affected NP and PA employment and training. According to the refined program theory, the first step in a decision to train or employ an NP or PA is participants' familiarity with, and medical doctors' trust in, the professions. This is followed by motivation—because deployment or training aligns with organisational aims—, and minimal perceived barriers to training or employment. Policies that did not affect these mechanisms among decision‐making participants had little to no influence on employment and training.

#### 5.2.1 Familiarity and Trust

Familiarity and positive experiences were a main reason for NP and PA training and employment for 22% of the respondents (Table 3). Key incentives were organisational or colleague experience with the professions (PA 57%/27%, NP 78%/30%, respectively), and an NP and/or PA working group (PA 25%, NP 31%) (Appendix S2). Unfamiliarity, especially in intellectual disability services, was identified as a barrier for 22% of the respondents (Table 4). Intellectual disability physicians more often saw ‘lack of familiarity’ as an inhibiting factor (*χ* ^2^ (1) = 6.67, *p* = 0.01).

#### 5.2.2 Motivation

Motivational factors were classified under the following categories: (1) healthcare improvement, (2) investing in prevention, (3) offering career perspectives, (4) and investing in education and training. For 18% of the respondents, a perceived lack of need for change was a main reason not to employ or train NPs and PAs.

78% of the respondents cited healthcare improvement as a key factor for NP and PA training and employment. Managers and directors of hospital departments (*χ* ^2^ (1) = 5.662, *p* = 0.02) and specialised medical doctors in hospitals (independent *χ* ^2^ (1) = 4.420, *p* = 0.049, other *χ* ^2^ (1) = 4.542, *p* = 0.036) more frequently saw ‘healthcare improvement’ as a motivating factor. Specific incentives were grouped into: care organisation and processes, (cost) efficiency, quality, continuity, and accessibility.

Stimulating factors concerning care organisation and processes were: coordinating/directing roles (PA 16%, NP 41%), and projects/innovation execution (PA 18%, NP 36%). In primary, (nursing) home, and intellectual disability services, collaboration with other healthcare organisations was also mentioned (PA 9%, NP 37%). Regarding (cost) efficiency, 54% of respondents who considered ‘funding’ a main stimulus cited lower salary costs compared to specialised medical doctors, particularly in hospital care (PA 60%, NP 43%). Efficiency through deploying the right professional in the right role was another stimulus (PA 38%, NP 56%). Quality‐of‐care‐related drivers for employment and training included: a focus on specific patient groups (PA 34%, NP 65%), increasing patient satisfaction (PA 23%, NP 40%), and allowing more time for communication with patients and families (19% PA, 47% NP). Continuity of care was mentioned as a stimulus to NP and PA employment and training by 42% and 54% of the respondents, respectively. Improvement of care accessibility was also indicated as a stimulus in (nursing) home care, and intellectual disability services (resp. PA 5/54 (9%) and 8/45 (18%), NP 20%/36%).

32% and 10% of the respondents who selected ‘healthcare improvement’ as a primary motivation for NP and PA employment and training specifically indicated investing in prevention as a driver. Respondents who indicated ‘healthcare professionals’ labor market as a key driver noted providing career perspectives for higher education graduates as a stimulus (PA 28%, NP 44%), particularly for NPs in (nursing) home care (59%). Respondents emphasizing ‘education and training’ as a main stimulus indicated investing in educating healthcare professionals (PA 9/28 (32%), NP 64%).

#### 5.2.3 Perceived Barriers

Barriers were categorized as follows: (1) financial and efficiency, (2) education and training, (3) quality concerns, and (4) medical doctors' position and work. Although barriers were similar across sectors, some were more profound in specific sectors. Significant differences existed between medical doctors and other respondents.

Funding issues were prominent in primary and hospital care (48% and 41%). Hospital respondents who perceived funding as a main barrier, noted uncertainty about salary costs allocation between medical specialist companies and hospitals (36%). In hospitals, nursing home care, and intellectual disability services, uncertainty about staffing allocation (medical versus nursing staff) was perceived as a barrier (respectively PA 27%, 2/13 (15%), 2/19 (11%), and NP 27%, 9/13 (69%), 3/19 (16%)). In primary care, concerns included inadequate funding and uncertainty about salary costs allocation between day practices and out‐of‐hours services (PA 10/29 (34%), 6/29 (21%), NP 55%, 8/29 (28%)). In (nursing) home and intellectual disability services, health insurer regulations were barriers (PA 2/13 (15%), 5/19 (26%), NP 8/13 (62%), 7/19 (37%), respectively). Across all sectors, (future) funding uncertainty had hindered NP and PA employment and training (PA 28%, NP 38%). Specialised medical doctors within hospitals more frequently saw ‘funding’ as a barrier compared to other respondents (independent *χ* ^2^ (1) = 10.35, *p* = 0.002, other *χ* ^2^ (1) = 6.97, *p* = 0.01), while elderly care physicians and intellectual disability physicians viewed it less so (respectively, *χ* ^2^ (1) = 5.09, *p* = 0.03 and *χ* ^2^ (1) = 5.57, *p* = 0.02). 31% of primary care respondents, and 14% of all sectors, indicated practical issues as a reason for not hiring and training (see 5.4.).

15% of the respondents perceived ‘education and training’ as a barrier, noting time investment and administrative burden (PA 25%, NP 33%). Primary care respondents indicated a wider range of (sector‐specific) training barriers than other sectors, including uncertainty about future grants (PA 4/14 (29%), NP 5/14 (36%)), competition with medical residents (PA 2/14 (14%), NP 3/14 (21%)), no solution for general practitioners who want to train NPs and PAs but do not want to employ them afterward (PA 1/14 (7%), NP 3/14 (21%)), and small practices having limited financial capacity to train (PA 2/14 (14%), NP 2/14 (14%)).

Quality concerns were cited by 11% of the respondents as barriers, including doubts about care quality (PA 57%, NP 53%), competencies (PA 43%, NP 41%), and patient satisfaction (PA 22%, NP 8/51 (16%)). Specialized medical doctors in hospitals more frequently indicated quality concerns (*χ* ^2^ (1) = 11.59, *p* = 0.002). Additional barriers noted by six respondents in hospital, primary, and (nursing) home care included doctors' concerns about the impact of NPs and PAs on medical doctors' position and work, as well as effects on doctors in training.

### 5.3 Government NP/PA Policies (Intervention)

Respondents mentioned several policies as stimuli for NP and PA employment and training. These policies were clustered into: (1) removing professional practice restrictions, (2) facilitating cost‐effective training and deployment, (3) providing knowledge on deployment, training, and healthcare outcomes, and (4) establishing sectoral agreements. Granting graduates an MSc degree was not mentioned as a stimulus.

Among respondents who indicated policy as a stimulus, respondents viewed expanding the scope of practice as an incentive for hiring and training PAs (57%) and NPs (76%). Legal acknowledgment was mentioned as a stimulus by 41% (PAs) and 45% (NPs). Among funding interventions, reimbursement regulations in hospital care stimulated employment and training, by allowing NPs and PAs to independently register and bill for healthcare services (PA 39%, NP 43%), as well as offering performance and billing options for co‐treatment and intercollegial consultations (PA 31%, NP 34%).

Respectively, 64% and 57% of the respondents who indicated ‘education and training’ as a main stimulus mentioned training grants as an incentive for NP and PA training. However, respondents across healthcare sectors reported insufficient training positions (PA 27%, NP 49%) and uncertainty about future grants (PA 18%, NP 23%) as barriers. In primary care, the training grants were too low according to, respectively, 6 and 3 of 14 respondents in this group (PA 21%, NP 43%).

In primary care, 5% of the respondents cited the Task Shifting Knowledge Center as influencing NP and PA training and employment decisions. In hospital care, two respondents mentioned healthcare outcome research as a stimulus. Sectoral agreements on NP and PA apprenticeships were noted as incentives in hospital and primary care (PA 5/27 (19%) and 1/4 (25%), NP 5/27 (19%) and 2/4 (50%)).

### 5.4 Under What Circumstances (Context)

Circumstances affected how decision‐making participants interpreted and acted on policies, functioning both as stimuli and barriers for NP and PA hiring and training.

#### 5.4.1 Flanking Policies

Various policies regarding healthcare system change were indicated as stimuli for NP and PA employment and training. Examples included the transition of healthcare from secondary to shared‐care services and primary care in hospital, primary, and (nursing) home care (PA 7/43 (16%), NP 37%). Population screenings and network medicine were stimuli in hospital and primary care (resp. PA 13/11%, NP 15/26%). In hospital care, the 2015 transition from tariffs consisting of a hospital component and a fee component for self‐employed medical specialists to integrated funding served as a stimulus (22%). In nursing home care, stimuli were an increase in financial leeway through quality framework/sector‐specific funds and the policy of living at home longer (respectively PA 1–1/11 (9%), NP 1–7/30 (3/23%)). Three respondents in hospital care cited the influence of healthcare budgets.

#### 5.4.2 Stakeholders

Policies of governments, umbrella organisations, or healthcare organisations were motivators for NP and PA employment and training for 13% of the respondents. Key factors included organisational vision and policies (39% PA, 63% NP), professional associations' task shifting documents (43% PA, 55% NP), and care protocols and guidelines (PA 8/49 (16%), NP 27%). Health insurers' funding opportunities were incentives according to 3–5 of 15 primary care respondents who indicated funding as a main stimulus (20%–33%).

6% of the respondents indicated that policies are a main barrier to hiring or training NPs and PAs. Primary care barriers included a lack of agreements and uncertainty about task delineation within umbrella organisations and scientific medical associations (PA 38%, NP 45%). In primary, (nursing) home, and intellectual disability services, poor alignment of education with the healthcare sector was reported (PA 5/41 (12%), NP 27%). Primary care respondents stated that training facilitation was less well‐supported than that for general practitioners (PA 1/14 (7%), NP 2/14 (14%)).

#### 5.4.3 Labor Market

61% of the respondents mentioned that labor market conditions encouraged NP and PA training and employment. Reasons included a high specialised medical doctor workload (43% PAs, 54% NPs), shortages of specialised medical doctors (PA resp. 30%, NP 41%), and limited specialist training capacity for medical doctors (20% PAs, 15% NPs). Hospital care respondents mentioned that NPs and PAs were employed to substitute for medical doctors in high‐turnover roles (e.g., ward doctors) to improve care continuity (PA 35%, NP 21%). General practitioners cited the labor market more frequently as a stimulus (*χ* ^2^ (1) = 5.628, *p* = 0.025). They mentioned general practitioner workload and shortages (PA 55/47%, NP 66/53%), and succession problems (PA 42%, NP 37%).

14% of the respondents (25% in intellectual disability services) indicated the labor market as a main barrier. Respondents noted a shortage of NPs and PAs (45/19%) with sectoral experience (45/21%). Respectively, 42% and 19% experienced shortages of bachelor‐level professionals willing to train as NPs and PAs. Respondents noted limited time for supervising NP and PA students due to workload pressures (PA 19%, NP 43%).

#### 5.4.4 Healthcare Demand

42% cited changing healthcare demand as a driver for NP and PA training and employment. Stimuli were population aging and growth and increased comorbidity (PA 30%, NP 64%), higher societal demands and expectations (PA 13%, NP 38%), and a more complex population in intellectual disability services (PA 4/27 (15%), NP 89%). Specialised medical doctors within hospital departments more frequently saw demand changes as a motivator (*χ* ^2^ (1) = 4.542, *p* = 0.036). 4% of respondents indicated changing healthcare demand as a main barrier to hiring and training. Some mentioned that higher complexity and expectations required the deployment of a specialised medical doctor rather than an NP or PA (43% and 57% respectively).

#### 5.4.5 Healthcare Organisation Setting

According to respondents, organisational vision and policy stimulated employment and training (PAs 39%, NPs 63%). Specifically, in primary care and long‐term care, barriers included lack of agreements and uncertainty about task delineation within the healthcare organisation (PA 9/29 (31%), NP 48%). In primary care, 31% of the respondents noted practical impediments compared to 11%–12% in other healthcare sectors. These included limited time for training (PA 7/19 (37%), NP 8/19 (42%)), limited financial resources (3/19 (16%)), employer responsibilities (PA 3/19 (16%), NP 2/19 (11%)), and insufficient treatment rooms and facilities (PA 10/19 (53%), NP 7/19 (37%)). General practitioners significantly more often reported practical barriers (*χ* ^2^ (1) = 18.25, *p* = 0.00).

### 5.5 Participants in the Decision‐Making Process (Actors)

**FIGURE 5:**
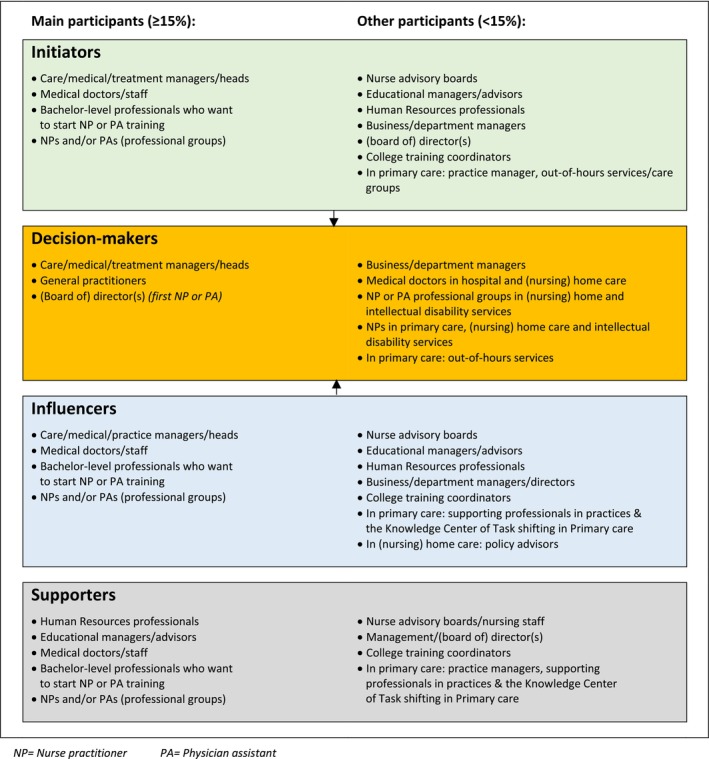
Participants and their roles in the decision‐making process.

Figure 5 depicts four key roles in the NP/PA employment and training decision‐making process: initiators, influencers, supporters, and decision‐makers. Survey data showed that main initiators included care/medical/treatment managers or heads, directors, medical doctors, NP/PA professional groups, and bachelor‐level professionals pursuing NP or PA training. All except directors also acted as influencers. Human Resources staff, educational advisors, medical doctors, bachelor‐level professionals, NPs, and PAs were identified as supporters without direct influence.

Final decision‐makers varied by sector, but were mainly care/medical/treatment/department managers or heads, or—when employing the first NP/PA—directors. In primary care, general practitioners made the final decisions. Various participants were mentioned by fewer than 15% of the respondents. NPs were sometimes involved as decision‐makers outside hospital care. In primary care, external parties occasionally supported or influenced decisions.

### 5.6 Outcome

In 2019, 64% and 75% of respondents expected more PAs and NPs in their work setting within 5 years, with 19% and 23% anticipating an increase of 25% or more (Appendix S2). Between 2019 and 2024, NP/PA FTEs rose by 150% and 181% in hospital care, 188% and 275% in primary care, and 180% and 556% in (nursing) home care. In intellectual disability services, NP FTEs increased by 250%, and PA numbers rose from an unrecorded baseline to 11. The average PA training program intake increased by 26% across all sectors. NP intake increased by 17%, except in hospital care (Advisory Committee on Health Workforce Planning 2025).

NP growth was more frequently anticipated in (nursing) home care (*χ* ^2^ (1) = 18.51, *p* = 0.00). Respondents in hospital care more often expected an increase in the number of PAs (*χ* ^2^ (1) = 34.36, *p* = 0.00). Educational advisors in hospital care were significantly more likely to expect an increase in NPs and PAs (*χ* ^2^ (1) = 10.07, *p* = 0.00, *χ* ^2^ (1) = 4.61, *p* = 0.04). In intellectual disability services, respondents were more often uncertain about how the number of PAs and NPs would develop (*χ* ^2^ (1) = 26.73, *p* = 0.00, *χ* ^2^ (1) = 19.73, *p* = 0.00); yet business and care/medical managers significantly more often predicted a strong PA increase (*χ* ^2^ (1) = 7.12, *p* = 0.007, *χ* ^2^ (1) = 6.37, *p* = 0.03).

### 5.7 Patterns in How Government Policies Led to Various Sector Outcomes

The scale of NP and PA deployment and training (outcome) varied widely across healthcare sectors, despite largely similar policies (intervention). To identify patterns in circumstances, participants, and mechanisms likely leading to these varied results, we used retroduction and an Intervention‐Context‐Actor‐Mechanism‐Outcome matrix (Appendix S3). Sectoral differences in NP and PA employment and training largely stemmed from decision‐maker types and sector‐specific and organisational circumstances.

In *primary care*, NP and PA deployment and training intake were (relatively) low in 2019 (2.1/1.0 per 100 general practitioner FTEs, and 31 and 24 annually). Respondents expected an increase in NPs (70%) and PAs (57%) within 5 years. Although there were many drivers for NP and PA deployment, financial and practical barriers limited employment and training. Many barriers related to the relatively small size of practices: limited financial resources and insufficient treatment rooms or facilities. Also, respondents indicated insufficient funding for deployment and a broad range of training barriers. General practitioners' concerns about their work and position and care quality had a greater impact, as they were the main decision‐makers on NP and PA hiring and training. In other sectors, managers and directors made these decisions.

*Hospital care* had the highest annual training intake (232/170) and NP and PA deployment in absolute numbers. Compared to specialized medical doctors, deployment was average (7.8/4.4 per 100 FTEs). 75% and 73% of hospital care respondents expected an increase in NPs and PAs over the next 5 years. For PAs, this was higher than in other sectors. NP and PA deployment and training were medium to high due to high familiarity and trust, numerous motivating factors concerning healthcare improvement, and few experienced barriers.

In (*nursing*) *home care* and *intellectual disability services*, NP deployment was high and PA deployment low (NP 26.8/15.4, PA 1.9/insufficient for calculation per 100 specialised medical doctor FTEs). The average annual training intake was 55 and 3 (NPs) and 7 and 0 (PAs). NP growth was expected by 92% ((nursing) home care) and 65% (intellectual disability services), and PA growth by 69% and 39%, respectively. Fewer barriers were experienced than in primary and hospital care. Labor market challenges were drivers for NP and PA hiring and training. This primarily involved shortages and workload concerns for specialised medical doctors, and the desire to offer career perspectives for bachelor‐level professionals. Unfamiliarity—especially with PAs—remained a major hurdle. Furthermore, the labor market was more frequently a concern in intellectual disability services, particularly regarding NP and PA shortages.

## 6 Discussion

This study presents a refined and verified program theory across Dutch healthcare sectors, showing how and why government policies and sectoral and organisational circumstances influenced NP and PA employment and training. By identifying the mechanisms at play, our findings expand previous work on skill mix and task shifting policies and provide actionable insights for tailoring policies to specific healthcare settings. Such policies are increasingly recognized as part of the solution to maintaining accessible, high‐quality healthcare, considering an impending gap between care demand and supply.

The impact of government policies on NP and PA employment and training varied significantly across healthcare sectors, shaped by circumstances. The results revealed more variation in context and decision‐makers than anticipated in the refined program theory. Specific primary care circumstances hampered the deployment of NPs and PAs.

Key sectoral factors included flanking policies, stakeholder support, labor market conditions, and healthcare demand. Organisational factors—policies, resources, aims, and whether the main decision‐makers were medical doctors or managers/directors—also played a critical role. The findings underscored that context is at least as influential as government policies, highlighting the importance of tailoring policies to specific healthcare settings.

### 6.1 How and Why NP/PA Policies Work (Or Not)

The findings confirmed that Dutch government policies facilitated NP and PA employment and training by expanding the scope of practice, legally acknowledging the professions, providing training grants, enabling billing rights, funding evaluation research, establishing a sectoral task shifting knowledge center, and facilitating sectoral agreements on apprenticeships. These measures enhanced familiarity and trust, removed barriers, and increased motivation among decision‐makers.

Consistent with previous studies (Dankers‐de Mari, van Vught, et al. 2023), scope of practice expansion and training grants emerged as strong incentives for NP and PA hiring and training. The expansion facilitated task shifting by increasing deployment opportunities by removing legal barriers. NPs and PAs gained an advantage over bachelor‐level professionals by being authorized to perform reserved medical procedures independently. This reduced supervision, lowered medical doctors' workload, and enabled management to organize care delivery more efficiently (Dankers‐de Mari, Thijssen, et al. 2023; de Bruijn‐Geraets et al. 2018). Training grants lowered financial barriers. However, their impact was limited in primary care, where small practice size, insufficient training positions, and uncertain future grants discouraged investment in training (Kouwen et al. 2020; van der Biezen et al. 2017).

Additionally, we verified the impact of reimbursement regulations, legal acknowledgment, evaluation research, and a task‐shifting knowledge center. Reimbursement regulations reduced financial barriers to deployment while motivating decision‐makers to deploy NPs and PAs. Decision‐makers recognized financial advantages over bachelor‐level professionals. Further, they saw opportunities for relieving administrative work and enabling outpatient clinic redesigns. Legal acknowledgment removed a barrier in organisations where it was a prerequisite for deployment by Human Resources. Furthermore, respondents confirmed the importance of sector‐specific interventions, such as evaluation research and a sectoral task‐reallocation knowledge center. These helped in fostering familiarity and enhancing medical doctors' trust (Dankers‐de Mari, Thijssen, et al. 2023).

Sectoral agreements on targeted numbers of NP and PA apprenticeships were added to the program theory. They likely contributed to increasing familiarity among decision‐making participants and medical doctors' trust in the professions, and their motivation to employ and train NPs and PAs to meet the agreed targets. Furthermore, we added the importance of long‐term policies, as uncertainty about future regulations and funding was frequently cited as a barrier. A lack of information complicates decision‐making and amplifies the aversion to risking invested time and resources (Ben‐Haim 2006).

### 6.2 The Influence of Circumstances on NP and PA Hiring and Training

Our results align with previous qualitative research on the significant impact of organisational and sectoral circumstances on NP and PA deployment (Niezen and Mathijssen 2014; Pittman et al. 2020; Pittman et al. 2021; Poghosyan, Nannini, Stone, and Smaldone 2013; Yee et al. 2013). Larger organisations with semi‐autonomous departments, such as hospitals, offered more favourable conditions than small primary care practices, which often lacked the resources to support training. This supports Greenhalgh et al.'s (2004) argument that organisational size is a proxy for readiness to assimilate innovations (Greenhalgh et al. 2004). Trust and familiarity also proved decisive. Echoing prior work (Niezen and Mathijssen 2014), lack of trust among physicians and unfamiliarity with NPs and PAs were significant barriers to NP/PA training and employment in intellectual disability services.

### 6.3 Participants in the Decision‐Making Process for NP and PA Hiring and Training

The survey revealed a broader range of actors in the decision‐making process than previously identified (Dankers‐de Mari, Thijssen, et al. 2023; Lovink et al. 2019; Timmermans et al. 2016; van der Biezen et al. 2017). NPs had greater influence on decision‐making than previously indicated, particularly in long‐term care.

Variation in NP and PA employment and training partly reflected whether final decisions rested with managers and directors or medical doctors. In primary care, general practitioners often dominated decisions. Their concerns about their work, position, or care quality were barriers to NP and PA deployment (Dankers‐de Mari, Thijssen, et al. 2023). This aligns with Freidson's theory of professions, which states that physicians control the content of their work and labor division as the dominant profession in medicine (Freidson 1970), and Abbott (1988) who states that professionals are interrelated and constantly interact, competing to define and control their expertise (Abbott 1988).

### 6.4 Strengths and Limitations

A key strength of this study is the broad applicability of the theoretical framework for the development and implementation of NP/PA or skill mix policies across countries and professions. The study surveyed a large, diverse group of respondents across healthcare sectors.

Due to the lack of validated questionnaires suited to our research objectives and the Dutch context, we developed interview and survey instruments based on the initial program theory (Dankers‐de Mari et al. 2021). Investigator‐developed questionnaires lack standardisation, which limits comparability with other studies and may introduce bias in question phrasing (Rowley 2014). To ensure neutral wording, the questionnaires were developed in collaboration with stakeholders.

To mitigate limitations of online surveys, such as the inability to capture emotions and to identify causal relationships (Patten 2017; Queiros et al. 2017), survey data were used solely to refine and test findings from a previous interview study and a longitudinal multimethod study (Dankers‐de Mari, Thijssen, et al. 2023; Dankers‐de Mari, van Vught, et al. 2023). Combining surveys and interviews allowed the study to benefit from the complementary strengths of both methods (Patten 2017; Queiros et al. 2017; Rowley 2014).

Literature on structural determinants of innovativeness assumes that determinants can be treated as variables whose impact can be isolated and independently quantified (Greenhalgh et al. 2004). Our realist‐inspired theory acknowledges that outcomes depend on how actors interpret and act on interventions, depending on their context. The added value of our study lies in understanding the underlying mechanisms at play, offering insights into how and why interventions will work (or not) across contexts.

A limitation was the low response rate of 10%. However, the high number of respondents may mitigate such limitation. A considerable number of respondents discontinued participation after viewing the introduction page with detailed study information. This may be explained by some sectoral and professional associations circulating a shortened invitation text, causing individuals to realize only upon reading the introduction page that they were not part of the target population. Furthermore, selection bias may have underrepresented critical perspectives, as some networks were positively inclined toward NP and PA hiring and training. However, respondents noted more barriers than drivers for hiring and training.

Moreover, while the results led to refinement and verification of components of the refined program theory, they did not lead to falsification. Also, despite extensive prior literature and interview research and surveying a broad and diverse group of respondents, critics might argue that by combining NPs and PAs into one theory we are painting with a broad brush. However, the decision‐making process and influencing factors are highly similar for NPs and PAs, and probably for other relatively new healthcare professions, as supported by previous research (Pittman et al. 2020).

Some macro‐level factors included in the refined program theory, such as healthcare budgets and evaluation studies, were rarely mentioned. However, respondents did cite related meso‐level factors, such as departmental budgets and protocols and guidelines, which are shaped by macro‐level policies. In the hybrid Dutch healthcare system, with both public and private elements, government influence is often indirect, mediated through funding (Maarse and Jeurissen 2024).

### 6.5 Recommendations for Further Research

We encourage researchers to further test the refined and verified program theory and configurations as presented in Figure 4 and Appendix S1, using realist methodology (Greenhalgh et al. 2011; Wong et al. 2017). Testing in other settings and on other professions will strengthen both the generalizability and applicability and will also deepen the insight into causal relationships and the interplay between contextual factors. Because in different groups different mechanisms operate in different contexts, policies will generate different outcomes. However, we expect the clusters of circumstances and the underlying mechanisms to be similar. In addition, we recommend further research into the impact of actor characteristics (micro level) on decision‐making.

### 6.6 Implications for Policy and Practice

Future Dutch policies should focus on increasing familiarity with and trust in NPs and PAs in primary and long‐term care. Options include sectoral evaluation research and task shifting knowledge centers. Further, policies should address perceived barriers by: (1) providing adequate training grants in primary care, (2) ensuring sufficient grant‐funded training positions, (3) further exploration of reimbursement and scope of practice options (preceded by pilots and physician consultation to enhance trust), and (4) encouraging training programs to align education with non‐hospital settings. Primary care policies should also address practical challenges, such as accommodating expanding primary care practices. Long‐term, sector‐specific policies with clarity on future funding are essential.

The theoretical framework is likely applicable to other countries and professions, with anticipated variation in components, such as decision‐makers and healthcare systems. We developed and applied the program theory to the Dutch health system, but we should recognize that this system differs from those of other countries. The Dutch Ministry of Health, Welfare and Sport oversees the stewardship, planning, and regulation of the health system, without a hierarchical relationship with payers or providers. Policy development typically occurs through cross‐sector stakeholder collaboration (Kroneman et al. 2024). This increases the influence of Dutch stakeholders, such as health insurers and sectoral and professional organisations. Health system differences across countries lead to variation in context, which will affect the decision‐making process and therefore outcomes in NP/PA deployment and training. However, policymakers, healthcare funders, organisations, and professionals can use the theoretical framework to anticipate mechanisms, tailor policies to specific settings, and draw inspiration from the policies discussed. In doing so, the program theory facilitates the design of effective NP, PA, and skill‐mix policies, which contributes to maintaining accessible, high‐quality healthcare in response to the widening care gap.

## 7 Conclusion

This study presents a refined and verified program theory showing how and why Dutch government policies stimulated NP and PA employment and training, and how sectoral and organisational circumstances shaped their impact. The findings underscore that the effectiveness of policies significantly depended on these circumstances, highlighting the importance of tailoring policies to specific healthcare settings.

Scope of practice expansion and training grants emerged as strong incentives for the hiring and training of NPs and PAs. In addition, reimbursement regulations, legal acknowledgment, evaluation research, sector‐specific knowledge centers for task shifting, and sectoral agreements facilitated their employment and training. Policies had an impact by enhancing familiarity and trust, increasing motivation, and removing perceived barriers among decision‐makers. Long‐term, sector‐specific policies that provide clarity on future funding are essential to support NP and PA deployment, especially in primary and long‐term care, to maintain accessible, high‐quality healthcare.

## Author Contributions

All authors have agreed on the final version and meet at least one of the following criteria (recommended by the ICMJE): (1) substantial contributions to conception and design, acquisition of data, or analysis and interpretation of data; (2) drafting the article or revising it critically for important intellectual content. **Ronald Batenburg:** formal analysis, conceptualization, methodology, validation, writing – review and editing. **Ellen J. C. M. Dankers‐de Mari:** conceptualization, methodology, validation, formal analysis, investigation, resources, data curation, writing – original draft, writing – review and editing, visualisation, supervision, project administration. **Patrick P. T. Jeurissen:** conceptualization, methodology, validation, writing – review and editing. **Anneke J. A. H. van Vught:** conceptualization, methodology, validation, writing – review and editing.

## Funding

The data were collected within the research program of the Advisory Committee on Health Workforce Planning (“Stichting Capaciteitsorgaan” in Dutch). No external funding was received.

## Conflicts of Interest

The authors declare no conflicts of interest.

## Supporting information


**Appendix S1:** jan70433‐sup‐0001‐AppendixS1.docx.

**Appendix S2:** jan70433‐sup‐0002‐AppendixS2.docx.

**Appendix S3:** jan70433‐sup‐0003‐AppendixS3.docx.

**Appendix S4:** jan70433‐sup‐0004‐AppendixS4.docx.

**Appendix S5:** jan70433‐sup‐0005‐AppendixS5.docx.

## Data Availability

Data openly available in a public repository that issues datasets with DOIs.
