# Supplementary material for: How Government Policies and Organisational and Sectoral Circumstances Influence Nurse Practitioner and Physician Assistant Employment and Training: A Realist Analysis Using Surveys
Source: J Adv Nurs. 2025 Dec 15;82(8):7900–16. doi: 10.1111/jan.70433 (PMC13356431; doi:10.1111/jan.70433)
Supplement: Supplementary file 1 — Appendix S1: jan70433‐sup‐0001‐AppendixS1.docx. [file JAN-82-7900-s005.docx]

###### Appendix A Detailed conceptual representation refined and verified ICAMO-configurations on NP and PA employment and training

##

Labor market

Stakeholders

Healthcare demand

Flanking policies

**Intervention**

NP and PA policy program

1. medical (specialist) doctor, resident, NP/PA, bachelor level professional capacity
2. physician continuity
3. *Image health-care sector*

- training grants
- reimbursement regulations
- funding knowledge center & evaluation research
- expanding scope of practice
- legal acknowledgement professions *& educational level*
- sectoral NP/PA agreements

1. health insurers support and reimbursement
2. support professional associations on deployment *& visibility*
3. university training facilitation
4. healthcare demand and complexity
5. expecta-tions from society
6. Resident capacity policies
7. healthcare system change
8. population screenings
9. integrated and additional funding
10. healthcare budgets

##

##

Context - macro

Outcomes - macro

**Actors**’ decision-maker type and characteristics:

- NP/PA experience
- *training attitude*
- medical doctors’ concerns
- medical doctor workload

Healthcare organization

Scale of employment & training in healthcare organizations

1. organizational support, vision & policy on NP/PA deployment *and visibility*, and (un)clarity on salary costs/staffing/task allocation;
2. organizational aims to: healthcare improvement, invest in prevention, offer career opportunities, and invest in education;
3. care/cure complexity, presence/absence of protocols/guidelines, extramural care, clearly/ill-defined conditions/patient groups;
4. organizational size and resources

- number of NP/PAs
- foreseen growth NP/PA capacity

Outcomes - meso

Context - meso

**Familiarity & Trust**

**Motivation**

**r**

**Perceived barriers**

Mechanisms

*Underlined text= refinements Cursive text = not falsified/verified in surveys NP= Nurse practitioner PA= Physician assistant ICAMO= Intervention-Context-Actor-Mechanism-Outcome*
