# Supplementary material for: How Government Policies and Organisational and Sectoral Circumstances Influence Nurse Practitioner and Physician Assistant Employment and Training: A Realist Analysis Using Surveys
Source: J Adv Nurs. 2025 Dec 15;82(8):7900–16. doi: 10.1111/jan.70433 (PMC13356431; doi:10.1111/jan.70433)
Supplement: Supplementary file 2 — Appendix S2: jan70433‐sup‐0002‐AppendixS2.docx. [file JAN-82-7900-s003.docx]

## Appendix B Tables

This appendix includes tables detailing the stimuli and barriers for hiring and/or training NPs and PAs and the expected development of the number of NP/PAs deployed within the own work setting over the next five years. Tests for significant differences across sectors and respondent types are available upon request.

#### Stimuli for hiring and/or training NPs and/or PAs

Healthcare improvement

*Table. Reasons / stimulating factors for hiring and/or training PAs and NPs regarding healthcare improvement*

| **PAs** | **Hospital care** | | | | **Primary care** | | | **(Nursing) home care** | | | **Intellectual disability services** | | | **Total** | | |
| --- | --- | --- | --- | --- | --- | --- | --- | --- | --- | --- | --- | --- | --- | --- | --- | --- |
|  | **n** | | **%** | | **n** | **%** | | **n** | **%** | | **n** | **%** | | **n** | **%** | |
| Ensuring continuity of care | 90 | | 54% | | 10 | 34% | | 10 | 19% | | 13 | 29% | | **123** | **42%** | |
| Deploying the right professional in the right role | 84 | | 50% | | 10 | 34% | | 8 | 15% | | 11 | 24% | | **113** | **38%** | |
| Improve healthcare organization | 78 | | 46% | | 9 | 31% | | 8 | 15% | | 10 | 22% | | **105** | **35%** | |
| Focus on specific patient groups | 73 | | 43% | | 8 | 28% | | 9 | 17% | | 10 | 22% | | **100** | **34%** | |
| Focus on specific domains (for example wound or palliative care) | 56 | | 33% | | 4 | 14% | | 4 | 7% | | 11 | 24% | | **75** | **25%** | |
| Increase patient satisfaction | 49 | | 29% | | 5 | 17% | | 7 | 13% | | 7 | 16% | | **68** | **23%** | |
| Execute projects/innovations | 41 | | 24% | | 4 | 14% | | 3 | 6% | | 4 | 9% | | **52** | **18%** | |
| Fulfilling a coordinating/directing role | 35 | | 21% | | 6 | 21% | | 4 | 7% | | 3 | 7% | | **48** | **16%** | |
| Investing in prevention | 13 | | 8% | | 3 | 10% | | 3 | 6% | | 11 | 24% | | **30** | **10%** | |
| Improve quality of life | - | | - | | - | - | | 7 | 13% | | 10 | 22% | | **17** | **-** | |
| Deployment at locations with limited capacity elderly care or intellectual disability physicians | - | | - | | - | - | | 5 | 9% | | 12 | 27% | | **17** | **-** | |
| Improve accessibility of care | - | | - | | - | - | | 5 | 9% | | 8 | 18% | | **13** | **-** | |
| Professional development of other healthcare professionals | - | | - | | - | - | | 5 | 9% | | 8 | 18% | | **13** | **-** | |
| Collaboration with other healthcare organizations | - | | - | | 2 | 7% | | 1 | 2% | | 9 | 20% | | **12** | **9%** | |
| Need for nursing/medical knowledge | - | | - | | - | - | | - | - | | 10 | 22% | | **10** | **-** | |
| Connection with neighborhood | - | | - | | 4 | 14% | | - | - | | - | - | | **4** | **-** | |
| Being able to discuss with colleagues | - | | - | | 3 | 10% | | - | - | | - | - | | **3** | **-** | |
| Involvement in community nursing | - | | - | | - | - | | 0 | 0% | | - | - | | **0** | **-** | |
| **NPs** | |  | | |  | | |  | | |  | | |  | | |
| Focus on specific patient groups | | 110 | | 65% | 21 | | 72% | 30 | | 56% | 31 | | 69% | **192** | | **65%** |
| Deploying the right professional in the right role | | 84 | | 50% | 23 | | 79% | 32 | | 59% | 28 | | 62% | **167** | | **56%** |
| Focus on specific domains (for example wound or palliative care) | | 90 | | 54% | 16 | | 55% | 29 | | 54% | 31 | | 69% | **166** | | **56%** |
| Ensuring continuity of care | | 87 | | 52% | 16 | | 55% | 30 | | 56% | 27 | | 60% | **160** | | **54%** |
| Improve healthcare organization | | 84 | | 50% | 14 | | 48% | 29 | | 54% | 30 | | 67% | **157** | | **53%** |
| Fulfilling a coordinating/directing role | | 75 | | 45% | 14 | | 48% | 18 | | 33% | 15 | | 33% | **122** | | **41%** |
| Increase patient satisfaction | | 68 | | 40% | 16 | | 55% | 16 | | 30% | 19 | | 42% | **119** | | **40%** |
| Execute projects/innovations | | 55 | | 33% | 11 | | 38% | 25 | | 46% | 17 | | 38% | **108** | | **36%** |
| Investing in prevention | | 41 | | 24% | 9 | | 31% | 18 | | 33% | 28 | | 62% | **96** | | **32%** |
| Deployment at locations with limited capacity elderly care or intellectual disability physicians | | - | | - | - | | - | 20 | | 37% | 28 | | 62% | **48** | | **-** |
| Collaboration with other healthcare organizations | | - | | - | 9 | | 31% | 13 | | 24% | 25 | | 56% | **47** | | **37%** |
| Improve quality of life | | - | | - | - | | - | 19 | | 35% | 27 | | 60% | **46** | | **-** |
| Professional development of other healthcare professionals | | - | | - | - | | - | 13 | | 24% | 27 | | 60% | **40** | | **-** |
| Need for nursing/medical knowledge | | - | | - | - | | - | - | | - | 30 | | 67% | **30** | | **-** |
| Improve accessibility of care | | - | | - | - | | - | 11 | | 20% | 16 | | 36% | **27** | | **-** |
| Being able to discuss with colleagues | | - | | - | 9 | | 31% | - | | - | - | | - | **9** | | **-** |
| Connection with neighborhood | | - | | - | 8 | | 28% | - | | - | - | | - | **8** | | **-** |
| Involvement in community nursing | | - | | - | - | | - | 3 | | 6% | - | | - | **3** | | **-** |
| **Number of respondents in this cluster** | | **168** | |  | **29** | |  | **54** | |  | **45** | |  | **296** | |  |

Healthcare labor market

*Table. Reasons / stimulating factors for hiring and/or training PAs and NPs regarding the healthcare labor market*

| **PAs** | **Hospital care** | | **Primary care** | | **(Nursing) home care** | | **Intellectual disability services** | | **Total** | |
| --- | --- | --- | --- | --- | --- | --- | --- | --- | --- | --- |
|  | **n** | **%** | **n** | **%** | **n** | **%** | **n** | **%** | **n** | **%** |
| Workload specialized medical doctors | 54 | 51% | 21 | 55% | 9 | 18% | 15 | 41% | **99** | **43%** |
| Shortage in the labor market for specialized medical doctors | 27 | 25% | 18 | 47% | 9 | 18% | 15 | 41% | **69** | **30%** |
| Providing career prospects for higher education graduates | 42 | 40% | 6 | 16% | 9 | 18% | 7 | 19% | **64** | **28%** |
| Limited capacity AIOS (physicians training for specialization) | 38 | 36% | 3 | 8% | 1 | 2% | 5 | 14% | **47** | **20%** |
| Limited AIOS deployment regarding continuity | 37 | 35% | - | - | - | - | - | - | **37** | **-** |
| Workload AIOS (physicians training for specialization) | 26 | 25% | - | - | 2 | 4% | 2 | 5% | **30** | **-** |
| Workload other healthcare professionals | 13 | 12% | 9 | 24% | 4 | 8% | 4 | 11% | **30** | **13%** |
| Shortage in the labor market for other healthcare professionals (in related fields) | 10 | 9% | 6 | 16% | 3 | 6% | 6 | 16% | **25** | **11%** |
| Desire to work less among medical specialists or district nurses | 9 | 8% | 9 | 24% | 2 | 4% | 1 | 3% | **21** | **9%** |
| Succession issues in general practice care | - | - | 16 | 42% | - | - | - | - | **16** | **-** |
| Deployment at locations with no or limited capacity of specialized medical doctors | - | - | - | - | 2 | 4% | 11 | 30% | **13** | **-** |
| Guidance for career switchers | - | - | - | - | 0 | 0% | 4 | 11% | **4** | **-** |
| Flexible deployment in fluctuating work conditions | 1 | 1% | 1 | 3% | 0 | 0% | 1 | 3% | **3** | **1%** |
| Workload district nurses | - | - | - | - | 0 | 0% | - | - | **0** | **-** |
| Shortage in the labor market for district nurses | - | - | - | - | 0 | 0% | - | - | **0** | **-** |
| **NPs** |  | |  | |  | |  | |  | |
| Workload specialized medical doctors | 48 | 45% | 25 | 66% | 28 | 55% | 24 | 65% | **125** | **54%** |
| Providing career prospects for higher education graduates | 49 | 46% | 10 | 26% | 30 | 59% | 13 | 35% | **102** | **44%** |
| Shortage in the labor market for specialized medical doctors | 19 | 18% | 20 | 53% | 30 | 59% | 25 | 68% | **94** | **41%** |
| Workload other healthcare professionals | 15 | 14% | 9 | 24% | 12 | 24% | 7 | 19% | **43** | **19%** |
| Shortage in the labor market for other healthcare professionals (in related fields) | 15 | 14% | 11 | 29% | 8 | 16% | 7 | 19% | **41** | **18%** |
| Limited capacity AIOS (physicians training for specialization) | 20 | 19% | 4 | 11% | 6 | 12% | 4 | 11% | **34** | **15%** |
| Deployment at locations with no or limited capacity of specialized medical doctors | - | - | - | - | 10 | 20% | 14 | 38% | **24** | **-** |
| Workload AIOS (physicians training for specialization) | 14 | 13% | - | - | 6 | 12% | 3 | 8% | **23** | **-** |
| Limited AIOS deployment regarding continuity | 22 | 21% | - | - | - | - | - | - | **22** | **-** |
| Desire to work less among medical specialists or district nurses | 5 | 5% | 8 | 21% | 5 | 10% | 1 | 3% | **19** | **8%** |
| Succession issues in general practice care | - | - | 14 | 37% | - | - | - | - | **14** | **-** |
| Flexible deployment in fluctuating work conditions | 1 | 1% | 1 | 3% | 0 | 0% | 1 | 3% | **3** | **1%** |
| Workload district nurses | - | - | - | - | 2 | 4% | - | - | **2** | **-** |
| Shortage in the labor market for district nurses | - | - | - | - | 2 | 4% | - | - | **2** | **-** |
| Guidance for career switchers | - | - | - | - | - | - | 2 | 5% | **2** | **-** |
| **Number of respondents in this cluster** | **106** |  | **38** |  | **51** |  | **37** |  | **232** |  |

Healthcare demand

*Table. Reasons / stimulating factors for hiring and/or training PAs and NPs regarding healthcare demand*

| **PAs** | **Hospital care** | | **Primary care** | | **(Nursing) home care** | | **Intellectual disability services** | | **Total** | |
| --- | --- | --- | --- | --- | --- | --- | --- | --- | --- | --- |
|  | **n** | **%** | **n** | **%** | **n** | **%** | **n** | **%** | **n** | **%** |
| Aging population, population growth, and/or increase in comorbidity | 28 | 37% | 9 | 35% | 2 | 7% | - | - | **39** | **30%** |
| The right care at the right place | 30 | 40% | 6 | 23% | 1 | 3% | - | - | **37** | **28%** |
| More time for communication with patients and their loved ones | 20 | 27% | 4 | 15% | 3 | 10% | 3 | 11% | **30** | **19%** |
| Transition of care from secondary to shared-care services, primary, and/or community care | 19 | 25% | 6 | 23% | 1 | 3% | - | - | **26** | **20%** |
| Higher demands/expectations from society | 11 | 15% | 3 | 12% | 3 | 10% | 3 | 11% | **20** | **13%** |
| Population screening(s) | 12 | 16% | 1 | 4% | - | - | - | - | **13** | **-** |
| Network medicine | 9 | 12% | 2 | 8% | - | - | - | - | **11** | **-** |
| More patients per standard practice | - | - | 5 | 19% | - | - | - | - | **5** | **-** |
| More complex patient population | - | - | - | - | - | - | 4 | 15% | **4** | **-** |
| Need for 24/7 care | - | - | 3 | 12% | - | - | 0 | 0% | **3** | **-** |
| Ambulatory care | - | - | 2 | 8% | 0 | 0% | - | - | **2** | **-** |
| Changing practice population (e.g., SES, ethnicity) | - | - | 2 | 8% | - | - | - | - | **2** | **-** |
| Living at home longer | - | - | - | - | 1 | 3% | - | - | **1** | **-** |
| Future role in community nursing | - | - | - | - | 0 | 0% | - | - | **0** | **-** |
| **NPs** |  | |  | |  | |  | |  | |
| Aging population, population growth, and/or increase in comorbidity | 47 | 63% | 19 | 73% | 18 | 60% | - | - | **84** | **64%** |
| The right care at the right place | 42 | 56% | 16 | 62% | 16 | 53% | - | - | **74** | **56%** |
| More time for communication with patients and their loved ones | 41 | 55% | 6 | 23% | 15 | 50% | 12 | 44% | **74** | **47%** |
| Transition of care from secondary to shared-care services, primary, and/or community care | 34 | 45% | 13 | 50% | 17 | 57% | - | - | **64** | **49%** |
| Higher demands/expectations from society | 23 | 31% | 7 | 27% | 13 | 43% | 17 | 63% | **60** | **38%** |
| Network medicine | 17 | 23% | 9 | 35% | 0 | - | - | - | **26** | **-** |
| More complex patient population | - | - | - | - | - | - | 24 | 89% | **24** | **-** |
| Population screening(s) | 14 | 19% | 1 | 4% | - | - | - | - | **15** | **-** |
| Ambulatory care | - | - | 6 | 23% | 5 | 17% | - | - | **11** | **-** |
| Living at home longer | - | - | - | - | 7 | 23% | - | - | **7** | **-** |
| Changing practice population (e.g., SES, ethnicity) | - | - | 7 | 27% | - | - | - | - | **7** | **-** |
| More patients per standard practice | - | - | 7 | 27% | - | - | - | - | **7** | **-** |
| Need for 24/7 care | - | - | 5 | 19% | - | - | - | - | **5** | **-** |
| Future role in community nursing | - | - | - | - | 2 | 7% | - | - | **2** | **-** |
| **Number of respondents in this cluster** | **75** |  | **26** |  | **30** |  | **27** |  | **158** |  |

Funding

*Table. Reasons / stimulating factors for hiring and/or training PAs and NPs regarding funding*

| **PAs** | **Hospital care** | | **Primary care** | | **(Nursing) home care** | | **Intellectual disability services** | | **Total** | |
| --- | --- | --- | --- | --- | --- | --- | --- | --- | --- | --- |
|  | **n** | **%** | **n** | **%** | **n** | **%** | **n** | **%** | **n** | **%** |
| Lower salary costs than specialized medical doctors | 40 | 60% | 8 | 53% | 4 | 36% | 3 | 33% | **55** | **54%** |
| Cost efficiency | 31 | 46% | 5 | 33% | 1 | 9% | 3 | 33% | **40** | **39%** |
| Independently register provided care (open/close Diagnosis Treatment Combinations) | 26 | 39% | - | - | - | - | - | - | **26** | **-** |
| Execution/declaration of co-treatment and intercollegial consultation | 21 | 31% | - | - | - | - | - | - | **21** | **-** |
| Introduction of integral funding | 15 | 22% | - | - | - | - | - | - | **15** | **-** |
| Regulations/agreements with health insurers/offices | 11 | 16% | 0 | 0% | 1 | 9% | 0 | 0% | **12** | **12%** |
| Cost distribution between medical specialist companies and hospital | 11 | 16% | - | - | - | - | - | - | **11** | **-** |
| Possibility of funding from health insurers’ innovation funds | - | - | 5 | 33% | - | - | - | - | **5** | **-** |
| Other funding possibilities | - | - | 5 | 33% | - | - | - | - | **5** | **-** |
| Contribution to entrepreneurial risk | 3 | 4% | - | - | - | - | - | - | **3** | **-** |
| Restriction on hiring freelancers | 0 | 0% | 0 | 0% | 3 | 27% | 0 | 0% | **3** | **3%** |
| Possibility of funding from health insurers' duty of care | - | - | 3 | 20% | - | - | - | - | **3** | **-** |
| Possibility of funding from health insurers’ elderly care module | - | - | 3 | 20% | - | - | - | - | **3** | **-** |
| More financial leeway through quality frameworks/sectorplus funds | - | - | - | - | 1 | 9% | - | - | **1** | **-** |
| **NPs** |  | |  | |  | |  | |  | |
| Lower salary costs than specialized medical doctors | 29 | 43% | 10 | 67% | 8 | 73% | 8 | 89% | **55** | **54%** |
| Cost efficiency | 27 | 40% | 6 | 40% | 6 | 55% | 9 | 100% | **48** | **47%** |
| Independently register provided care (open/close Diagnosis Treatment Combinations) | 29 | 43% | - | - | - | - | - | - | **29** | **-** |
| Execution/declaration of co-treatment and intercollegial consultation | 23 | 34% | - | - | - | - | - | - | **23** | **-** |
| Introduction of integral funding | 15 | 22% | - | - | - | - | - | - | **15** | **-** |
| Regulations/agreements with health insurers/offices | 11 | 16% | 0 | 0% | 2 | 18% | 2 | 22% | **15** | **15%** |
| Cost distribution between medical specialist companies and hospital | 13 | 19% | - | - | - | - | - | - | **13** | **-** |
| Possibility of funding from health insurers’ elderly care module | - | - | 5 | 33% | - | - | - | - | **5** | **-** |
| Possibility of funding from health insurers' duty of care | - | - | 4 | 27% | - | - | - | - | **4** | **-** |
| Possibility of funding from health insurers’ innovation funds | - | - | 4 | 27% | - | - | - | - | **4** | **-** |
| Other funding possibilities | - | - | 4 | 27% | - | - | - | - | **4** | **-** |
| Contribution to entrepreneurial risk | 3 | 4% | - | - | - | - | - | - | **3** | **-** |
| Restriction on hiring freelancers | 0 | 0% | 0 | 0% | 3 | 27% | 0 | 0% | **3** | **3%** |
| More financial leeway through quality frameworks/sectorplus funds | - | - | - | - | 1 | 9% | - | - | **1** | **-** |
| **Number of respondents in this cluster** | **67** |  | **15** |  | **11** |  | **9** |  | **102** |  |

Policy

*Table. Reasons / stimulating factors for hiring and/or training PAs and NPs regarding policy*

| **PAs** | **Hospital care** | | **Primary care** | | **(Nursing) home care** | | **Intellectual disability services** | | **Total** | |
| --- | --- | --- | --- | --- | --- | --- | --- | --- | --- | --- |
|  | **n** | **%** | **n** | **%** | **n** | **%** | **n** | **%** | **n** | **%** |
| Independent authority for reserved medical procedures / extending SOP | 18 | 67% | 2 | 50% | 4 | 33% | 4 | 67% | **28** | **57%** |
| Task shifting document – implementation guide | 15 | 56% | 2 | 50% | 1 | 8% | 3 | 50% | **21** | **43%** |
| Inclusion in the Individual Health Care Professions (IHCP) Act / legal acknowledgement | 12 | 44% | 2 | 50% | 2 | 17% | 4 | 67% | **20** | **41%** |
| Vision and policy of the healthcare organization | 10 | 37% | 2 | 50% | 3 | 25% | 4 | 67% | **19** | **39%** |
| Care at the right place | 6 | 22% | 2 | 50% | 0 | 0% | - | - | **8** | **-** |
| Care protocols or guidelines | 6 | 22% | 0 | 0% | 1 | 8% | 1 | 17% | **8** | **16%** |
| Transition of care from secondary to shared-care services and primary care | 5 | 19% | 1 | 25% | 1 | 8% | - | - | **7** | **16%** |
| Sectoral agreements about apprenticeships | 5 | 19% | 1 | 25% | - | - | - | - | **6** | **-** |
| Network medicine | 3 | 11% | 0 | 0% | - | - | - | - | **3** | **-** |
| Insightful tools for PA/NP deployment | 3 | 11% | - | - | - | - | - | - | **3** | **-** |
| Implementation of the Care and Compulsion Act (Wet zorg en dwang) | - | - | - | - | 0 | 0% | 1 | 17% | **1** | **-** |
| Vision and policy of care groups, health centers, or HAGRO (General Practitioner Partnerships) | - | - | 1 | 25% | - | - | - | - | **1** | **-** |
| Healthcare Quality, Complaints, and Disputes Act (WKKGZ): delineation of tasks/responsibilities | - | - | - | - | 0 | 0% | - | - | **0** | **-** |
| Shortened hospital stay durations | - | - | 0 | 0% | - | - | - | - | **0** | **-** |
| **NPs** |  | |  | |  | |  | |  | |
| Independent authority for reserved medical procedures / extending SOP | 18 | 67% | 2 | 50% | 11 | 92% | 6 | 100% | **37** | **76%** |
| Vision and policy of the healthcare organization | 13 | 48% | 2 | 50% | 10 | 83% | 6 | 100% | **31** | **63%** |
| Task shifting document – implementation guide | 14 | 52% | 2 | 50% | 7 | 58% | 4 | 67% | **27** | **55%** |
| Inclusion in the Individual Health Care Professions (IHCP) Act / legal acknowledgement | 11 | 41% | 2 | 50% | 4 | 33% | 5 | 83% | **22** | **45%** |
| Transition of care from secondary to shared-care services and primary care | 8 | 30% | 2 | 50% | 6 | 50% | - | - | **16** | **37%** |
| Care at the right place | 7 | 26% | 2 | 50% | 4 | 33% | - | - | **13** | **-** |
| Care protocols or guidelines | 6 | 22% | 0 | 0% | 5 | 42% | 2 | 33% | **13** | **27%** |
| Sectoral agreements about apprenticeships | 5 | 19% | 2 | 50% | - | - | - | - | **7** | **-** |
| Network medicine | 6 | 22% | 1 | 25% | - | - | - | - | **7** | **-** |
| Insightful tools for PA/NP deployment | 4 | 15% | 0 | 0% | - | - | - | - | **4** | **-** |
| Implementation of the Care and Compulsion Act (Wet zorg en dwang) | - | - | - | - | - | - | 2 | 33% | **2** | **-** |
| Shortened hospital stay durations | - | - | 1 | 25% | - | - | - | - | **1** | **-** |
| Healthcare Quality, Complaints, and Disputes Act (WKKGZ): delineation of tasks/responsibilities | - | - | - | - | 0 | 0% | 0 | 0% | **0** | **-** |
| Vision and policy of care groups, health centers, or HAGRO (General Practitioner Partnerships) | - | - | 0 | 0% | - | - | - | - | **0** | **-** |
| **Number of respondents in this cluster** | **27** |  | **4** |  | **12** |  | **6** |  | **49** |  |

Education and training

*Table. Reasons / stimulating factors for hiring and/or training PAs and NPs regarding education/training*

| **PAs** | **Hospital care** | | **Primary care** | | **(Nursing) home care** | | **Intellectual disability services** | | **Total** | |
| --- | --- | --- | --- | --- | --- | --- | --- | --- | --- | --- |
|  | **n** | **%** | **n** | **%** | **n** | **%** | **n** | **%** | **n** | **%** |
| Training grants for PA/NPs | 12 | 80% | 2 | 50% | 1 | 20% | 1 | 25% | **16** | **57%** |
| Training an NP/PA in-house provides a tailored healthcare professional | 7 | 47% | 3 | 75% | 0 | 0% | 0 | 0% | **10** | **36%** |
| Investing in education/training | 5 | 33% | 2 | 50% | 1 | 20% | 1 | 25% | **9** | **32%** |
| Shorter training duration compared to medical specialists | 3 | 20% | 2 | 50% | 0 | 0% | 1 | 25% | **6** | **21%** |
| Deployability during training | 5 | 33% | 1 | 25% | 0 | 0% | 0 | 0% | **6** | **21%** |
| Fewer training positions for specialized medical doctors | 3 | 20% | - | - | - | - | - | - | **3** | **-** |
| Individualization of training duration for medical doctors in specialist training (AIOS) | 3 | 20% | - | - | 0 | 0% | - | - | **3** | **-** |
| Limited intake for training to become an intellectual disability or elderly care physician | - | - | - | - | 1 | 20% | 1 | 25% | **2** | **-** |
| Good collaboration (within) primary care in joint NP/PA training | - | - | 1 | 25% | 0 | 0% | - | - | **1** | **-** |
| Healthcare organization can profile itself as a training organization | - | - | - | - | - | - | 1 | 25% | **1** | **-** |
| Shorter training duration for NPs than PAs | 0 | 0% | 0 | 0% | 0 | 0% | 0 | 0% | **0** | **0%** |
| Limited intake for training to become a district nurse | - | - | - | - | 0 | 0% | - | - | **0** | **-** |
| **NPs** |  | |  | |  | |  | |  | |
| Training grants for PA/NPs | 11 | 73% | 2 | 50% | 5 | 100% | 0 | 0% | **18** | **64%** |
| Investing in education/training | 9 | 60% | 2 | 50% | 5 | 100% | 2 | 50% | **18** | **64%** |
| Training an NP/PA in-house provides a tailored healthcare professional | 9 | 60% | 3 | 75% | 3 | 60% | 1 | 25% | **16** | **57%** |
| Deployability during training | 6 | 40% | 2 | 50% | 3 | 60% | 1 | 25% | **12** | **43%** |
| Shorter training duration compared to medical specialists | 3 | 20% | 1 | 25% | 2 | 40% | 2 | 50% | **8** | **29%** |
| Fewer training positions for specialized medical doctors | 3 | 20% | - | - | - | - | - | - | **3** | **-** |
| Individualization of training duration for medical doctors in specialist training (AIOS) | 2 | 13% | - | - | 1 | 20% | - | - | **3** | **-** |
| Limited intake for training to become an intellectual disability or elderly care physician | - | - | - | - | 3 | 60% | 0 | 0% | **3** | **-** |
| Shorter training duration for NPs than PAs | 0 | 0% | 1 | 25% | 1 | 20% | 0 | 0% | **2** | **7%** |
| Good collaboration (within) primary care in joint NP/PA training | - | - | 1 | 25% | 1 | 20% | - | - | **2** | **-** |
| Limited intake for training to become a district nurse | - | - | - | - | 1 | 20% | - | - | **1** | **-** |
| Healthcare organization can profile itself as a training organization | - | - | - | - | - | - | 1 | 25% | **1** | **-** |
| **Number of respondents in this cluster** | **15** |  | **4** |  | **5** |  | **4** |  | **28** |  |

Familiarity / positive experiences

*Table. Reasons / stimulating factors for hiring and/or training PAs and NPs regarding familiarity / positive experiences*

| **PAs** | **Hospital care** | | **Primary care** | | **(Nursing) home care** | | **Intellectual disability services** | | **Total** | |  |
| --- | --- | --- | --- | --- | --- | --- | --- | --- | --- | --- | --- |
|  | **n** | **%** | **n** | **%** | **n** | **%** | **n** | **%** | **n** | **%** |  |
| Experience with NP/PAs within the own healthcare organization | 38 | 79% | 3 | 43% | 4 | 24% | 2 | 20% | **47** | **57%** |  |
| Colleague experience with NP/PAs within other healthcare organizations or regions | 17 | 35% | 2 | 29% | 3 | 18% | 0 | 0% | **22** | **27%** |  |
| Practice examples through umbrella organizations or congresses | 9 | 19% | 0 | 0% | 1 | 6% | 0 | 0% | **10** | **12%** |  |
| Familiarity because of NP/PA working group | 18 | 38% | - | - | 1 | 6% | - | - | **19** | **-** |  |
| Familiarity because of NP/PA network | - | - | - | - | 0 | 0% | - | - | **0** | **-** |  |
| **NPs** |  | |  | |  | |  | |  | | |
| Experience with NP/PAs within the own healthcare organization | 35 | 73% | 5 | 71% | 15 | 88% | 9 | 90% | **64** | **78%** | |
| Colleague experience with NP/PAs within other healthcare organizations or regions | 16 | 33% | 2 | 29% | 5 | 29% | 2 | 20% | **25** | **30%** | |
| Practice examples through umbrella organizations or congresses | 10 | 21% | 1 | 14% | 1 | 6% | 2 | 20% | **14** | **17%** | |
| Familiarity because of NP/PA working group | 16 | 33% | - | - | 7 | 41% | - | - | **23** | **-** | |
| Familiarity because of NP/PA network | - | - | - | - | 2 | 12% | - | - | **2** | **-** | |
| **Number of respondents in this cluster** | **48** |  | **7** |  | **17** |  | **10** |  | **82** |  | |

#### Barriers to hiring and/or training NPs and/or PAs

Funding

*Table. Barriers to hiring and/or training PAs and NPs regarding funding*

| **PAs** | **Hospital care** | | **Primary care** | | **(Nursing) home care** | | **Intellectual disability services** | | **Total** | |
| --- | --- | --- | --- | --- | --- | --- | --- | --- | --- | --- |
|  | **n** | **%** | **n** | **%** | **n** | **%** | **n** | **%** | **n** | **%** |
| Distribution of costs between medical specialist companies and the hospital | 32 | 36% | - | - | - | - | - | - | **32** | **-** |
| Uncertainty about the distribution of salary costs | 25 | 28% | 6 | 21% | - | - | - | - | **31** | **-** |
| Uncertainty about staffing distribution between medical and nursing staff | 24 | 27% | - | - | 2 | 15% | 2 | 11% | **28** | **-** |
| Uncertainty about future reimbursement for PAs/NPs by health insurers | 23 | 26% | - | - | - | - | - | - | **23** | **-** |
| Uncertainty about future funding by health insurers | - | - | 11 | 38% | 2 | 15% | 6 | 32% | **19** | **-** |
| Inadequate funding by health insurers | - | - | 10 | 34% | - | - | - | - | **10** | **-** |
| Salary difference between PA/NP and bachelor level professionals | 7 | 8% | - | - | 1 | 8% | - | - | **8** | **-** |
| Potential higher pay scale (in the collective labor agreement) | - | - | 7 | 24% | - | - | - | - | **7** | **-** |
| Regulations or agreements with health insurers/offices | - | - | - | - | 2 | 15% | 5 | 26% | **7** | **-** |
| Higher salary costs than other professions such as bachelor level professionals or medical assistants | - | - | 3 | 10% | - | - | 2 | 11% | **5** | **-** |
| Uncertainty about current regulations from health insurers/offices | - | - | - | - | 2 | 15% | 2 | 11% | **4** | **-** |
| Uncertainty about future requirements for deployment from care offices | - | - | - | - | 2 | 15% | 2 | 11% | **4** | **-** |
| **NPs** |  | |  | |  | |  | |  | |
| Uncertainty about staffing distribution between medical and nursing staff | 24 | 27% | - | - | 9 | 69% | 3 | 16% | **36** | **-** |
| Uncertainty about future funding by health insurers | - | - | 16 | 55% | 9 | 69% | 8 | 42% | **33** | **-** |
| Distribution of costs between medical specialist companies and the hospital | 32 | 36% | - | - | - | - | - | - | **32** | **-** |
| Uncertainty about the distribution of salary costs | 20 | 22% | 8 | 28% | - | - | - | - | **28** | **-** |
| Uncertainty about future reimbursement for PAs/NPs by health insurers | 24 | 27% | - | - | - | - | - | - | **24** | **-** |
| Inadequate funding by health insurers | - | - | 16 | 55% | - | - | - | - | **16** | **-** |
| Regulations or agreements with health insurers/offices | - | - | - | - | 8 | 62% | 7 | 37% | **15** | **-** |
| Salary difference between PA/NP and bachelor level professionals | 6 | 7% | - | - | 5 | 38% | - | - | **11** | **-** |
| Uncertainty about current regulations from health insurers/offices | - | - | - | - | 7 | 54% | 4 | 21% | **11** | **-** |
| Higher salary costs than other professions such as bachelor level professionals or medical assistants | - | - | 4 | 14% | - | - | 5 | 26% | **10** | **-** |
| Potential higher pay scale (in the collective labor agreement) | - | - | 10 | 34% | - | - | - | - | **10** | **-** |
| Uncertainty about future requirements for deployment from care offices | - | - | - | - | 4 | 31% | 3 | 16% | **7** | **-** |
| **Number of respondents in this cluster** | **90** |  | **29** |  | **13** |  | **19** |  | **151** |  |

Lack of familiarity

*Table. Barriers to hiring and/or training PAs and NPs regarding lack of familiarity*

| **PAs** | **Hospital care** | | **Primary care** | | **(Nursing) home care** | | **Intellectual disability services** | | **Total** | |  |
| --- | --- | --- | --- | --- | --- | --- | --- | --- | --- | --- | --- |
|  | **n** | **%** | **n** | **%** | **n** | **%** | **n** | **%** | **n** | **%** |  |
| No experience with collaboration with NP/PA | 26 | 68% | 5 | 38% | 7 | 44% | 24 | 67% | **62** | **60%** |  |
| Limited or no insight into the consequences of hiring or training | 15 | 39% | 7 | 54% | 6 | 38% | 16 | 44% | **44** | **43%** |  |
| No experience with training NP/PAs | 12 | 32% | 4 | 31% | 6 | 38% | 20 | 56% | **42** | **41%** |  |
| Limited acceptance of NPs/PAs by other healthcare professionals | 14 | 37% | 3 | 23% | 5 | 31% | 7 | 19% | **29** | **28%** |  |
| Uncertainty about task delineation | 1 | 3% | 3 | 23% | 6 | 38% | 18 | 50% | **28** | **27%** |  |
| Uncertainty about authorities/competencies | 1 | 3% | 3 | 23% | 6 | 38% | 17 | 47% | **27** | **26%** |  |
| **NPs** |  | |  | |  | |  | |  | | |
| No experience with collaboration with NP/PA | 22 | 58% | 8 | 62% | 12 | 75% | 27 | 75% | **69** | **67%** | |
| Limited or no insight into the consequences of hiring or training | 14 | 37% | 9 | 69% | 8 | 50% | 17 | 47% | **48** | **47%** | |
| No experience with training NP/PAs | 8 | 21% | 5 | 38% | 10 | 63% | 23 | 64% | **46** | **45%** | |
| Uncertainty about task delineation | 1 | 3% | 3 | 23% | 11 | 69% | 20 | 56% | **35** | **34%** | |
| Limited acceptance of NPs/PAs by other healthcare professionals | 13 | 34% | 2 | 15% | 10 | 63% | 8 | 22% | **33** | **32%** | |
| Uncertainty about authorities/competencies | 1 | 3% | 2 | 15% | 11 | 69% | 19 | 53% | **33** | **32%** | |
| **Number of respondents in this cluster** | **38** |  | **13** |  | **16** |  | **36** |  | **103** |  | |

No need for change

*Table. Barriers to hiring and/or training PAs and NPs regarding ‘no need for change’*

| **PAs** | **Hospital care** | | | | **Primary care** | | | **(Nursing) home care** | | | **Intellectual disability services** | | | **Total** | | |
| --- | --- | --- | --- | --- | --- | --- | --- | --- | --- | --- | --- | --- | --- | --- | --- | --- |
|  | **n** | | **%** | | **n** | **%** | | **n** | **%** | | **n** | **%** | | **n** | **%** | |
| No need to expand the number of healthcare professionals | 24 | | 65% | | 5 | 56% | | 3 | 23% | | 14 | 50% | | **46** | **53%** | |
| No need to change the type of healthcare professionals | 18 | | 49% | | 5 | 56% | | 3 | 23% | | 17 | 61% | | **43** | **49%** | |
| NPs | |  | | |  | | |  | | |  | | |  | | |
| No need to expand the number of healthcare professionals | | 21 | | 57% | 7 | | 78% | 5 | | 38% | 17 | | 61% | **50** | | **57%** |
| No need to change the type of healthcare professionals | | 17 | | 46% | 6 | | 67% | 4 | | 31% | 21 | | 75% | **48** | | **55%** |
| **Number of respondents in this cluster** | | **37** | |  | **9** | |  | **13** | |  | **28** | |  | **87** | |  |

Healthcare labor market

*Table. Barriers to hiring and/or training PAs and NPs regarding the healthcare labor market*

| **PAs** | **Hospital care** | | **Primary care** | | **(Nursing) home care** | | **Intellectual disability services** | | **Total** | |
| --- | --- | --- | --- | --- | --- | --- | --- | --- | --- | --- |
|  | **n** | **%** | **n** | **%** | **n** | **%** | **n** | **%** | **n** | **%** |
| Shortage in the labor market of certified NPs/PAs with relevant work experience | 2 | 10% | 2 | 22% | 3 | 30% | 7 | 25% | **14** | **21%** |
| Limited capacity of bachelor's-level professionals who want to train as NPs/PAs | 7 | 35% | 0 | 0% | 1 | 10% | 5 | 18% | **13** | **19%** |
| Limited time for mentoring students due to high workload | 5 | 25% | 1 | 11% | 3 | 30% | 4 | 14% | **13** | **19%** |
| Shortage in the labor market of certified NPs/PAs | 3 | 15% | 1 | 11% | 3 | 30% | 6 | 21% | **13** | **19%** |
| Limited time for retraining NPs/PAs trained in other healthcare organizations | 3 | 15% | 0 | 0% | 2 | 20% | 3 | 11% | **8** | **12%** |
| Training an NP/PA reduces capacity for bachelor’s-level professionals | 2 | 10% | - | - | 0 | 0% | 0 | 0% | **2** | **-** |
| **NPs** |  | |  | |  | |  | |  | |
| Shortage in the labor market of certified NPs/PAs | 4 | 20% | 4 | 44% | 6 | 60% | 16 | 57% | **30** | **45%** |
| Shortage in the labor market of certified NPs/PAs with relevant work experience | 4 | 20% | 5 | 56% | 5 | 50% | 16 | 57% | **30** | **45%** |
| Limited time for mentoring students due to high workload | 7 | 35% | 6 | 67% | 7 | 70% | 9 | 32% | **29** | **43%** |
| Limited capacity of bachelor's-level professionals who want to train as NPs/PAs | 5 | 25% | 6 | 67% | 5 | 50% | 12 | 43% | **28** | **42%** |
| Limited time for retraining NPs/PAs trained in other healthcare organizations | 3 | 15% | 1 | 11% | 4 | 40% | 4 | 14% | **12** | **18%** |
| Training an NP/PA reduces capacity for bachelor’s-level professionals | 3 | 15% | - | - | 1 | 10% | 1 | 4% | **5** | **-** |
| **Number of respondents in this cluster** | **20** |  | **9** |  | **10** |  | **28** |  | **67** |  |

Practical

*Table. Barriers to hiring and/or training PAs and NPs regarding practical issues*

| **PAs** | **Hospital care** | | | | **Primary care** | | | | **(Nursing) home care** | | | **Intellectual disability services** | | | **Total** | | |
| --- | --- | --- | --- | --- | --- | --- | --- | --- | --- | --- | --- | --- | --- | --- | --- | --- | --- |
|  | **n** | | **%** | | **n** | | **%** | | **n** | **%** | | **n** | **%** | | **n** | **%** | |
| Insufficient treatment rooms or other facilities | 4 | | 15% | | 10 | | 53% | | 0 | 0% | | 1 | 8% | | **15** | **22%** | |
| A medical resident can provide coverage, but a PA or NP cannot | 9 | | 33% | | 4 | | 21% | | 0 | 0% | | 1 | 8% | | **14** | **21%** | |
| Perceived burden of being an employer | 4 | | 15% | | 3 | | 16% | | 1 | 11% | | 0 | 0% | | **8** | **12%** | |
| Limited support from the internal training coordinator | 5 | | 19% | | 0 | | 0% | | 1 | 11% | | 1 | 8% | | **7** | **10%** | |
| Limited time for training | - | | - | | 7 | | 37% | | - | - | | - | - | | **7** | **-** | |
| Limited financial resources for training | - | | - | | 3 | | 16% | | - | - | | - | - | | **3** | **-** | |
| Limited time to onboard a certified NP/PA | - | | - | | 3 | | 16% | | - | - | | - | - | | **3** | **-** | |
| Limited support from HRM | 2 | | 7% | | 0 | | 0% | | 0 | 0% | | 0 | 0% | | **2** | **3%** | |
| Limited financial resources to hire a certified NP/PA | - | | - | | 2 | | 11% | | - | - | | - | - | | **2** | **-** | |
| NPs | |  | | | |  | | |  | | |  | | |  | | |
| Insufficient treatment rooms or other facilities | | 4 | | 15% | | 7 | | 37% | 2 | | 22% | 3 | | 25% | **16** | | **24%** |
| A medical resident can provide coverage, but a PA or NP cannot | | 7 | | 26% | | 2 | | 11% | 1 | | 11% | 3 | | 25% | **13** | | **19%** |
| Limited support from the internal training coordinator | | 5 | | 19% | | 2 | | 11% | 2 | | 22% | 3 | | 25% | **12** | | **18%** |
| Perceived burden of being an employer | | 4 | | 15% | | 2 | | 11% | 3 | | 33% | 2 | | 17% | **11** | | **16%** |
| Limited time for training | | - | | - | | 8 | | 42% | - | | - | - | | - | **8** | | **-** |
| Limited support from HRM | | 2 | | 7% | | 0 | | 0% | 2 | | 22% | 2 | | 17% | **6** | | **9%** |
| Limited financial resources for training | | - | | - | | 3 | | 16% | - | | - | - | | - | **3** | | **-** |
| Limited time to onboard a certified NP/PA | | - | | - | | 3 | | 16% | - | | - | - | | - | **3** | | **-** |
| Limited financial resources to hire a certified NP/PA | | - | | - | | 2 | | 11% | - | | - | - | | - | **2** | | **-** |
| **Number of respondents in this cluster** | | **27** | |  | | **19** | |  | **9** | |  | **12** | |  | **67** | |  |

Quality of care

*Table. Barriers to hiring and/or training PAs and NPs regarding quality of care*

| **PAs** | **Hospital care** | | | | **Primary care** | | | | **(Nursing) home care** | | | **Intellectual disability services** | | | **Total** | | |  |
| --- | --- | --- | --- | --- | --- | --- | --- | --- | --- | --- | --- | --- | --- | --- | --- | --- | --- | --- |
|  | **n** | | **%** | | **n** | | **%** | | **n** | **%** | | **n** | **%** | | **n** | **%** | |  |
| Doubt about maintaining the quality of care | 16 | | 53% | | 4 | | 57% | | 4 | 50% | | 5 | 83% | | **29** | **57%** | |  |
| Doubt about competencies | 16 | | 53% | | 2 | | 29% | | 2 | 25% | | 2 | 33% | | **22** | **43%** | |  |
| Doubt about maintaining patient satisfaction | 4 | | 13% | | 3 | | 43% | | 1 | 13% | | 3 | 50% | | **11** | **22%** | |  |
| Lack of or limited scientific evidence regarding deployment in healthcare sector | 4 | | 13% | | 1 | | 14% | | 1 | 13% | | 2 | 33% | | **8** | **16%** | |  |
| Negative experience with NP/PA | 4 | | 13% | | 2 | | 29% | | 0 | 0% | | 0 | 0% | | **6** | **12%** | |  |
| **NPs** | |  | | | |  | | |  | | |  | | |  | | | |
| Doubt about maintaining the quality of care | | 12 | | 40% | | 4 | | 57% | 7 | | 88% | 4 | | 67% | **27** | | **53%** | |
| Doubt about competencies | | 10 | | 33% | | 4 | | 57% | 5 | | 63% | 2 | | 33% | **21** | | **41%** | |
| Doubt about maintaining patient satisfaction | | 1 | | 3% | | 3 | | 43% | 2 | | 25% | 2 | | 33% | **8** | | **16%** | |
| Lack of or limited scientific evidence regarding deployment in healthcare sector | | 1 | | 3% | | 2 | | 29% | 3 | | 38% | 2 | | 33% | **8** | | **16%** | |
| Negative experience with NP/PA | | 2 | | 7% | | 0 | | 0% | 3 | | 38% | 0 | | 0% | **5** | | **10%** | |
| **Number of respondents in this cluster** | | **30** | |  | | **7** | |  | **8** | |  | **6** | |  | **51** | |  | |

Education and training

*Table. Barriers to hiring and/or training PAs and NPs regarding education/training*

| **PAs** | **Hospital care** | | **Primary care** | | **(Nursing) home care** | | **Intellectual disability serv.** | | **Total** | |
| --- | --- | --- | --- | --- | --- | --- | --- | --- | --- | --- |
|  | **n** | **%** | **n** | **%** | **n** | **%** | **n** | **%** | **n** | **%** |
| Insufficient grant-funded training places | 13 | 41% | 4 | 29% | 2 | 14% | 1 | 8% | **20** | **27%** |
| Time investment/administrative burden of training | 9 | 28% | 4 | 29% | 5 | 36% | 0 | 0% | **18** | **25%** |
| Uncertainty about future grants for training NP/PAs | 6 | 19% | 4 | 29% | 1 | 7% | 2 | 15% | **13** | **18%** |
| Lack of knowledge or clarity about training grants | 4 | 13% | - | - | 2 | 14% | 2 | 15% | **8** | **11%** |
| Poor alignment of training with the healthcare sector | - | - | 0 | 0% | 2 | 14% | 3 | 23% | **5** | **7%** |
| Absence of retraining programs for NP/PAs trained elsewhere | 1 | 3% | 1 | 7% | 2 | 14% | 0 | 0% | **4** | **5%** |
| The grants for training are too low | - | - | 3 | 21% | - | - | - | - | **3** | **-** |
| Limited financial capacity to train within a small primary care practice | - | - | 2 | 14% | - | - | - | - | **2** | **-** |
| Few universities train for primary care | - | - | 2 | 14% | - | - | - | - | **2** | **-** |
| Shorter training duration NPs than PAs | 2 | 6% | 0 | 0% | 0 | 0% | 0 | 0% | **2** | **3%** |
| Competition with training of physicians training for specialization (AIOS) | - | - | 2 | 14% | - | - | - | - | **2** | **-** |
| The facilitation of training is not as good as with SBOH | - | - | 1 | 7% | - | - | - | - | **1** | **-** |
| Retraining an NP/PA trained elsewhere takes a lot of time | - | - | 1 | 7% | - | - | - | - | **1** | **-** |
| No solution for GPs who want to train an NP/PA but do not wish to employ them afterward | - | - | 1 | 7% | - | - | - | - | **1** | **-** |
| Training requires collaboration between the daytime practice and the out-of-hours GP service | - | - | 0 | 0% | - | - | - | - | **0** | **-** |
| Inability to offer a contract after completing the training | - | - | 0 | 0% | - | - | - | - | **0** | **-** |
| **NPs** |  | |  | |  | |  | |  | |
| Insufficient grant-funded training places | 13 | 41% | 6 | 43% | 9 | 64% | 8 | 62% | **36** | **49%** |
| Time investment/administrative burden of training | 9 | 28% | 6 | 43% | 8 | 57% | 1 | 8% | **24** | **33%** |
| Uncertainty about future grants for training NP/PAs | 5 | 16% | 5 | 36% | 3 | 21% | 4 | 31% | **17** | **23%** |
| Lack of knowledge or clarity about training grants | 4 | 13% | - | - | 4 | 29% | 5 | 38% | **13** | **18%** |
| Poor alignment of training with the healthcare sector | - | - | 4 | 29% | 2 | 14% | 5 | 38% | **11** | **15%** |
| Absence of retraining programs for NP/PAs trained elsewhere | 1 | 3% | 2 | 14% | 2 | 14% | 2 | 15% | **7** | **10%** |
| The grants for training are too low | - | - | 6 | 43% | - | - | - | - | **6** | **-** |
| Few universities train for primary care | - | - | 5 | 36% | - | - | - | - | **5** | **-** |
| Shorter training duration NPs than PAs | 3 | 9% | 0 | 0% | 2 | 14% | 0 | 0% | **5** | **7%** |
| No solution for general practitioners who want to train an NP/PA but do not wish to employ them afterward | - | - | 3 | 21% | - | - | - | - | **3** | **-** |
| Competition with training of physicians training for specialization (AIOS) | - | - | 3 | 21% | - | - | - | - | **3** | **-** |
| The facilitation of training is not as good as with SBOH | - | - | 2 | 14% | - | - | - | - | **2** | **-** |
| Retraining an NP/PA trained elsewhere takes a lot of time | - | - | 2 | 14% | - | - | - | - | **2** | **-** |
| Training requires collaboration between the daytime practice and the out-of-hours GP service | - | - | 2 | 14% | - | - | - | - | **2** | **-** |
| Limited financial capacity to train within a small primary care practice | - | - | 2 | 14% | - | - | - | - | **2** | **-** |
| Inability to offer a contract after completing the training | - | - | 0 | 0% | - | - | - | - | **0** | **-** |
| **Number of respondents in this cluster** | **32** |  | **14** |  | **14** |  | **13** |  | **73** |  |

Policy

*Table. Barriers to hiring and/or training PAs and NPs regarding policy*

| **PAs** | **Hospital care** | | **Primary care** | | **(Nursing) home care** | | **Intellectual disability services** | | **Total** | |
| --- | --- | --- | --- | --- | --- | --- | --- | --- | --- | --- |
|  | **n** | **%** | **n** | **%** | **n** | **%** | **n** | **%** | **n** | **%** |
| Uncertainty about authorities | 3 | 30% | 1 | 17% | 2 | 40% | 5 | 63% | **11** | **38%** |
| Lack of agreements/unclarity within umbrella organizations/scientific associations regarding task delineation | 5 | 50% | 2 | 33% | 1 | 20% | 3 | 38% | **11** | **38%** |
| Lack of agreements/clarity within the organization regarding task delineation | 2 | 20% | 1 | 17% | 2 | 40% | 4 | 50% | **9** | **31%** |
| Uncertainty about future government policy | 4 | 40% | 0 | 0% | 1 | 20% | 4 | 50% | **9** | **31%** |
| Unclear future policies of umbrella organizations/scientific associations | 4 | 40% | 0 | 0% | 1 | 20% | 2 | 25% | **7** | **24%** |
| Uncertainty about the positioning of the new profession 'practice nurse' | - | - | 1 | 17% | - | - | - | - | **1** | **-** |
| Lack of, or unclear vision, within the care group, health center, or HAGRO | - | - | 0 | 0% | - | - | - | - | **0** | **-** |
| No authority to determine death | - | - | 0 | 0% | - | - | - | - | **0** | **-** |
| **NPs** |  | |  | |  | |  | |  | |
| Uncertainty about authorities | 3 | 30% | 3 | 50% | 4 | 80% | 5 | 63% | **15** | **52%** |
| Lack of agreements/unclarity within the organization regarding task delineation | 3 | 30% | 2 | 33% | 4 | 80% | 5 | 63% | **14** | **48%** |
| Lack of agreements/clarity within umbrella organizations/scientific associations regarding task delineation | 5 | 50% | 4 | 67% | 2 | 40% | 2 | 25% | **13** | **45%** |
| Uncertainty about future government policy | 3 | 30% | 2 | 33% | 2 | 40% | 3 | 38% | **10** | **34%** |
| Unclear future policies of umbrella organizations/scientific associations | 3 | 30% | 1 | 17% | 1 | 20% | 2 | 25% | **7** | **24%** |
| Lack of, or unclear vision, within the care group, health center, or HAGRO | - | - | 1 | 17% | - | - | - | - | **1** | **-** |
| No authority to determine death | - | - | 1 | 17% | - | - | - | - | **1** | **-** |
| Uncertainty about the positioning of the new profession 'practice nurse' | - | - | 1 | 17% | - | - | - | - | **1** | **-** |
| **Number of respondents in this cluster** | **10** |  | **6** |  | **5** |  | **8** |  | **29** |  |

Healthcare demand

*Table. Barriers to hiring and/or training PAs and NPs regarding healthcare demand*

| **PAs** | **Hospital care** | | **Primary care** | | **(Nursing) home care** | | **Intellectual disability services** | | **Total** | |
| --- | --- | --- | --- | --- | --- | --- | --- | --- | --- | --- |
|  | **n** | **%** | **n** | **%** | **n** | **%** | **n** | **%** | **n** | **%** |
| Higher complexity and expectations require the involvement of a specialized medical doctor rather than an NP/PA | 7 | 70% | 1 | 33% | 2 | 67% | 2 | 40% | **12** | **57%** |
| Uncertainty about the pace of growth in healthcare demand | - | - | 0 | 0% | - | - | - | - | **0** | **-** |
| **NPs** |  | |  | |  | |  | |  | |
| Higher complexity and expectations require the involvement of a specialized medical doctor rather than an NP/PA | 4 | 40% | 1 | 33% | 1 | 33% | 3 | 60% | **9** | **43%** |
| Uncertainty about the pace of growth in healthcare demand | - | - | 0 | 0% | - | - | - | - | **0** | **-** |
| **Number of respondents in this cluster** | **10** |  | **3** |  | **3** |  | **5** |  | **21** |  |

#### Expected development of NP/PA deployment

*Table. Expected development of the number of PAs and NPs deployed within the own work setting over the next five years*

| **PAs** | **Hospital care** | | **Primary care** | | **(Nursing) home care** | | **Intellectual disability services** | | **Total** | |
| --- | --- | --- | --- | --- | --- | --- | --- | --- | --- | --- |
|  | **n** | **%** | **n** | **%** | **n** | **%** | **n** | **%** | **n** | **%** |
| Significant decrease (≤ -25%) | 5 | 3% | 3 | 7% | 1 | 2% | 1 | 1% | **10** | **3%** |
| Decrease (-5% to -25%) | 2 | 1% | 0 | 0% | 0 | 0% | 2 | 3% | **4** | **1%** |
| Slight decrease (up to -5%) | 1 | 1% | 1 | 2% | 0 | 0% | 1 | 1% | **3** | **1%** |
| Remain approximately the same | 33 | 18% | 9 | 21% | 7 | 17% | 20 | 26% | **69** | **20%** |
| Slight increase (up to 5%) | 29 | 15% | 0 | 0% | 10 | 24% | 8 | 11% | **47** | **14%** |
| Increase (5% to 25%) | 73 | 39% | 12 | 29% | 11 | 26% | 12 | 16% | **108** | **31%** |
| Significant increase (≥ 25%) | 36 | 19% | 12 | 29% | 8 | 19% | 10 | 13% | **66** | **19%** |
| I do not know | 9 | 5% | 5 | 12% | 5 | 12% | 22 | 29% | **41** | **12%** |
| **Total** | **188** |  | **42** |  | **42** |  | **76** |  | **348** |  |
| **NPs** |  | |  | |  | |  | |  | |
| Significant decrease (≤ -25%) | 3 | 2% | 3 | 6% | 0 | 0% | 3 | 3% | **9** | **2%** |
| Decrease (-5% to -25%) | 3 | 2% | 0 | 0% | 1 | 1% | 1 | 1% | **5** | **1%** |
| Slight decrease (up to -5%) | 4 | 2% | 2 | 4% | 0 | 0% | 2 | 2% | **8** | **2%** |
| Remain approximately the same | 26 | 14% | 5 | 9% | 4 | 5% | 13 | 13% | **48** | **11%** |
| Slight increase (up to 5%) | 33 | 18% | 3 | 6% | 14 | 18% | 19 | 18% | **69** | **16%** |
| Increase (5% to 25%) | 83 | 45% | 18 | 33% | 25 | 32% | 27 | 26% | **153** | **36%** |
| Significant increase (≥ 25%) | 23 | 12% | 17 | 31% | 33 | 42% | 22 | 21% | **96** | **23%** |
| I do not know | 10 | 5% | 6 | 11% | 1 | 1% | 17 | 16% | **34** | **8%** |
| **Total** | **184** |  | **54** |  | **78** |  | **104** |  | **422** |  |
