# Supplementary material for: How Government Policies and Organisational and Sectoral Circumstances Influence Nurse Practitioner and Physician Assistant Employment and Training: A Realist Analysis Using Surveys
Source: J Adv Nurs. 2025 Dec 15;82(8):7900–16. doi: 10.1111/jan.70433 (PMC13356431; doi:10.1111/jan.70433)
Supplement: Supplementary file 3 — Appendix S3: jan70433‐sup‐0003‐AppendixS3.docx. [file JAN-82-7900-s001.docx]

## Appendix C Intervention-Context-Actor-Mechanism-Outcome (ICAMO) matrix of distinctive elements healthcare sectors on NP and PA employment and training

|  | | **Hospital / medical specialist care*** | **Primary care** | **(Nursing) home care** | **Intellectual disability services** |
| --- | --- | --- | --- | --- | --- |
| **Intervention** | **Sectoral policies additional to the national NP/PA policy program** | reimbursement regulations evaluation research healthcare outcomes  sectoral agreement on apprenticeships | additional training grants  National Center of Knowledge for Task shifting in Primary Care  sectoral agreement on apprenticeships | upcoming reimbursement regulation extramural care | upcoming reimbursement regulation extramural care |
| **Circumstances** | **macro-level** *(healthcare sectors and nationally)* | Flanking policies:  transition of healthcare from secondary, to shared-care services and primary care  population screening  network medicine  integral funding  healthcare budgets  *generic training grants*  *medical doctor association support (personnel planning model)*  Stakeholders:  regulations from health insurers  *lack of support of some medical doctor associations*  Labor market:  resident capacity  medical doctor continuity  individualization (and shortening) training duration physicians in  specialist training | Flanking policies:  transition of healthcare from secondary, to shared-care services  and primary care  population screening  network medicine  Stakeholders:  funding opportunities health insurers  lack of agreements / uncertainty within umbrella organizations  and scientific associations about task delineation */ (previous) lack*  *of support general practitioner association*  training barriers (competition with medical residents, less well-  supported training facilitation)  poor alignment of training with healthcare sector  Labor market:  general practitioner workload  general practitioner shortages  succession problems  desire to work less hours among general practitioners  limited number of NP/PAs (with experience in the sector)  limited capacity of bachelor's level professionals willing to train as  NP/PAs | Flanking policies:  transition of healthcare from secondary, to shared-care services and primary care  quality framework /sector-specific funds in nursing home care  policy ‘living at home longer’  Stakeholders:  regulations from health insurers  NP nursing home network  lack of agreements / uncertainty within umbrella organizations  and scientific associations about task delineation and scope of  practice / *lack of support elderly care physician association*  (unclarity) health insurer regulations and funding / *absence*  *payment title*  poor alignment of training with healthcare sector  Labor market:  (deployment at locations with) limited physician capacity  limited number of NP/PAs (with experience in the sector)  limited capacity of bachelor's level professionals willing to train as  NP/PAs  *poor sectoral image* | Stakeholders:  regulations from health insurers  Healthcare demand:  a more complex patient population  Stakeholders:  lack of agreements / uncertainty within umbrella organizations  and scientific associations about task delineation and scope of  practice  (unclarity) health insurer regulations and funding  poor alignment of training with healthcare sector  Labor market:  (deployment at locations with) limited physician capacity  limited number of NP/PAs (with experience in the sector)  limited capacity of bachelor's level professionals willing to train as  NP/PAs  *poor sectoral image* |
|  | **meso-level** *(healthcare organizations)* | *less complex and protocoled cure/care*  *NP/PA visibility*  *organizational support*  unclarity about the allocation of salary costs / staffing between:  1) medical doctor companies and hospitals, and 2) between  medical and nursing staff  *lack of formation (budget)* | *organizational support out-of-hours practices/ care groups*  *complex care for bachelor level professionals*  lack of agreements / unclarity about task delineation within  healthcare organization  practical impediments: limited organizational size and resources  (treatment rooms/facilities)  funding: inadequate funding & unclarity about salary costs  allocation between day practices & out-of-hours posts | *complex care for bachelor level professionals*  *extramural care*  lack of agreements / unclarity about task delineation within  healthcare organization  unclarity or lack of agreements about staffing allocation between  medical and nursing staff  *high complexity and multimorbidity make task delineation difficult* | increase in care complexity (for bachelor level professionals)  *extramural care*  lack of agreements / unclarity about task delineation within  healthcare organization  unclarity or lack of agreements about staffing allocation between  medical and nursing staff |
| **Actors** | **main**  **decision-makers** | - managers - (board of) director(s) (first NP/PA) | - general practitioners | - care, medical, treatment heads - managers - (board of) director(s) (first NP/PA) | - care, medical, treatment heads - managers - (board of) director(s) (first NP/PA) |
| **Mechanisms** | **Familiarity & trust** | familiarity & trust | familiarity & trust | familiarity & trust | unfamiliarity |
|  | **Motivation** | improvement of care quality, organization, continuity, and  processes  cost efficiency (funding: independently register and bill healthcare  services) | improvement of continuity of care (labor market challenges:  general practitioner workload, shortages & succession problems)  cost efficiency (funding: by healthcare insurers) | improvement of care quality, organization, and processes  continuity and accessibility of care (labor market challenges:  elderly care physician workload & shortages, and offering career  perspectives) | improvement of care quality, organization, and processes  continuity and accessibility of care (labor market challenges:  intellectual disability physician workload & shortages, and offering  career perspectives) |
|  | **Perceived barriers** | financial (funding: uncertainty about allocation of salary  costs/staffing) | financial (inadequate & uncertain funding)  practical  education and training | - | labor market (shortages of NP/PAs (with experience in the  healthcare sector)  no need for change |
| **Outcome** | **macro-level**  **FTE NP-PA / per 100 FTE**  **medical doctor** | 2019: 1.423 FTE NPs and 813 FTE PAs / 7.8 – 4.4  2024: 2.130 FTE NPs (+150%) and 1.470 FTE PAs (+181%) | 2019: 197 FTE NPs and 91 FTE PAs / 2.1 – 1.0  2024: 370 FTE NPs (+188%) and 250 FTE PAs (+275%) | 2019: 389 FTE NPs and 27 FTE PAs / 26.8 – 1.9  2024: 700 FTE NPs (+180%) and 150 FTE PAs (+556%) | 2019: 32 FTE NPs and - FTE PAs / 15.4 - **  2024: 80 FTE NPs (+250%) and 11 FTE PAs |
|  | **macro-level**  **intake training program***** | 2015-2019: 232 MANP / 170 MPA  2020-2024: 216 MANP / 182 MPA | 2015-2019: 30 MANP / 24 MPA  2020-2024: 41 MANP / 54 MPA | 2015-2019: 58 MANP / 7 MPA  2020-2024: 100 MANP / 16 MPA | 2015-2019: 5 MANP / 0 MPA  2020-2024: 12 MANP / 1 MPA |
|  | **meso-level** (bandwidth 2019) | most 2-5 NPs (0 - 100)  most 2-5 PAs (0 - >100) | most 2-5 NPs (0 - >100)  most 0 PAs (0 - >100) | most 2-5 NPs (0 - 50)  most 0 PAs (0 - 5) | most 2-5 NPs (0 - 50)  most 0 PAs (0 - 5) |
|  | **meso-level** (expectation 2019-2024) | 75% expects increase in NPs, most 5-25%  73% expects increase in PAs, most 5-25% | 70% expects increase in NPs, most 5-25%  57% expects increase in PAs, most 5-25% & >25% | 92% expects increase in NPs, most > 25%  69% expects increase in PAs, most 5-25% | 65% expects increase in NPs, most 5-25%  39% expects increase in PAs, most expect capacity remains  stable |

** Including rehabilitation care / ** too small to calculate / *** 5-year average Cursive= not falsified/verified in surveys NP=Nurse practitioner PA=Physician assistant*
